# Prognostic Scores for Liver Resection in Colorectal Metastases: Performance, Limitations, and Methodological Pitfalls—A Systematic Review and Meta-Analysis

**DOI:** 10.3390/cancers18040625

**Published:** 2026-02-14

**Authors:** Luca Viganò, Luca Risi, Elisa Ragaini, Francesca Ieva, Elena Desiato

**Affiliations:** 1 Hepatobiliary Unit, Department of Minimally Invasive General & Oncologic Surgery, Humanitas Gavazzeni University Hospital, 24125 Bergamo, Italy; luca.risi@humanitas.it (L.R.); elisa.ragaini@gmail.com (E.R.); elena.desiato@humanitas.it (E.D.); 2 Department of Biomedical Sciences, Humanitas University, Pieve Emanuele, 20072 Milan, Italy; 3 MOX Laboratory, Department of Mathematics, Politecnico di Milano, 20133 Milan, Italy; francesca.ieva@polimi.it; 4 CHDS—Center for Health Data Science, Human Technopole, 20157 Milan, Italy

**Keywords:** prognostic scores, colorectal liver metastases, liver resection, prediction of survival, predicting performance, meta-analysis

## Abstract

Background/Objectives: The prediction of survival after resection of colorectal liver metastases is crucial for planning treatment strategies. Several prognostic scores have been proposed, but their reliability is debated. The present study aims to review available prognostic scores, focusing on their performance and the methodological approaches adopted for their evaluation. Methods: A systematic literature review was conducted using PubMed, Embase, and the Cochrane Database, including studies published between January 2015 and June 2024. Only English-language studies reporting the external validation of prognostic models were included. A random-effects meta-analysis was performed. Results: Overall, 48 prognostic scores were externally validated across 48 studies (*n* = 33,602 patients). A total of 286 performance measurements were reported, utilizing 17 different metrics and considering four outcomes: overall survival (OS), cancer-specific survival, recurrence-free survival (RFS), and recurrence rate. For OS, the pooled C-index values for the Fong, GAME, and RAS mutation Clinical Risk scores were 0.578 (0.570–0.587), 0.609 (0.592–0.625), and 0.579 (0.471–0.688), respectively. For RFS, the pooled C-index for the Fong score was 0.616 (0.578–0.653). Scores incorporating genetic, immunological and radiomic data performed better than purely clinical ones (C-index = 0.610, 0.657 and 0.635, respectively, vs. 0.585, *p* < 0.05). Analogously, the scores including perioperative data outperformed preoperative ones (C-index = 0.671 vs. 0.600, *p* = 0.007). Conclusions: The current survival prediction relies on scores with low reliability (C-index ≤ 0.65). Despite the abundance of available data, their heterogeneity and variable quality have limited their usability. Future research should prioritize the development of new prognostic tools and the standardization of prognostic modeling and reporting.

## Simple Summary

Survival prediction after liver resection for colorectal metastases is crucial for planning treatment strategies. A systematic review and meta-analysis of studies reporting the external validation of prognostic models for liver resection in colorectal metastases was performed. The current survival prediction relies on scores with limited reliability. Available data are heterogeneous, calling for standardization in reporting. Future studies should focus on development of new prognostic tools and prioritize the standardization of prognostic modeling and reporting.

## 1. Introduction

Colorectal cancer is the third most common cancer worldwide, accounting for over 1.9 million cases annually [1] and responsible for 9.3% of cancer-related deaths [2]. Approximately 15–25% of colorectal cancer patients present with liver metastases at diagnosis, while an additional 10–20% develop metastases during follow-up after primary tumor resection [3,4]. Over the past 40 years, surgery has proven to be an effective treatment for colorectal liver metastases (CRLM) and, since 2000, has been considered the standard of care whenever technically feasible [3,4,5,6]. Its effectiveness has been further enhanced by the adoption of perioperative systemic therapies, which allow for selecting candidates, downsizing the disease, and converting some patients from unresectability to resectability [3,7,8,9].

Despite the clear benefits of resection, accurate survival prediction after surgery remains essential for several reasons. First, the risk of post-surgical recurrence is high (over 50%), with 15–20% of patients experiencing early recurrence and early cancer-related death, deriving no benefit from hepatectomy [10,11]. Second, while preoperative systemic therapies are increasingly used, patients with favorable tumor biology may be suitable for upfront surgery without neoadjuvant chemotherapy [3,12]. Finally, alternatives to surgery are emerging, such as thermal ablation for small oligonodular disease and transplantation for patients with high tumor burden [13,14], with their adoption relying precisely on the comparative analysis of expected outcomes.

Since the 1990s, several prognostic scores have been developed for CRLM patients, with some, notably the Fong Score [15], RAS mutation Clinical Risk Score (RASmut-CRS) [16], and GAME score [17], being widely used in both clinical practice and scientific research. Despite their widespread application, the statistical performance of these scores varies significantly across different external validation studies and populations. Their reliability remains a subject of debate, and none is currently considered the standard. Moreover, in recent years, many authors have investigated new prognostic factors based on genetic, radiomic, and immunological data, suggesting that these approaches may outperform standard clinical predictive models.

The present study aims to perform a systematic review and meta-analysis of the performance of the available prognostic scores for patients undergoing surgery for CRLM, and to examine the quality of the existing evidence on this topic.

## 2. Materials and Methods

The present study was registered in the Prospective Register of Systematic Reviews (PROSPERO, registration number CRD42024599732) and was conducted according to the PRISMA guidelines (checklist available in Supplementary Table S1) [18].

The primary endpoint of the study was to evaluate, through a systematic review and meta-analysis of the literature, the performance at external validation of available prognostic models in predicting overall survival in patients with CRLM undergoing resection.

The secondary endpoints were: (1) to evaluate the performance at external validation of the models in predicting other survival outcomes (e.g., recurrence-free survival or early recurrence); (2) to analyze the methodology used for external validation, with particular focus on the homogeneity and adequacy of statistical approaches and data reporting; and (3) to assess the contribution of different types of predictors (e.g., clinical, genetic, or radiomic) to model performance.

### 2.1. Literature Search

A comprehensive literature search was conducted using three biomedical databases (MEDLINE, Embase, and the Cochrane Database). The final search was conducted for studies published between 1 January 2015 and 1 June 2024. The search used both controlled vocabulary (MeSH/Emtree) and free-text terms covering four concepts: colorectal liver metastases, surgery, survival, and prognostic models. We chose not to include terms related to ‘external validation’ or ‘score performance’ in the search strategy, as this could have risked missing articles that did not explicitly focus on these aspects (for example, studies developing a new score without external validation but comparing it with previously published scores, thereby providing external validation for the latter).

Inclusion criteria were as follows:

- English-language articles;
- Studies reporting at least one long-term outcome (i.e., overall survival, cancer-specific survival, recurrence-free survival, or recurrence rate at a specified time point);
- Studies analyzing the performance of prognostic scores (including scores, formulae, and nomograms);
- Studies reporting external validation of at least one prognostic score (either previously published scores validated in the study population or newly developed scores that were externally validated within the same study);
- Studies providing at least one measure of the statistical performance of the score.
- Exclusion criteria included:
- Studies involving patients undergoing palliative resection;
- Studies including patients with recurrent CRLM;
- Case reports, book chapters, reviews, meta-analyses, consensus statements, editorials, and conference abstracts.

If multiple publications originated from the same research group or institution with substantial overlap in aims (validated score) and study populations, only the publication with the largest cohort was included. Studies including patients undergoing resection combined with thermal ablation, or isolated thermal ablation, were considered only if the ablation group accounted for <15% of the entire cohort. Similarly, studies including both R0/R1 and R2 resections were considered only if the R2 subgroup represented <5% of the cohort.

### 2.2. Search Strategy

In 2019, He et al. published a systematic review on the performance of prognostic scores for colorectal cancer patients, both metastatic and non-metastatic, at external validation [19]. The topic is the same as in the present review, with the exception that He et al. also included non-metastatic patients and those with non-hepatic metastases. They used the same search strategy and query as the present review, with the only difference being the absence of restriction to liver metastases. Their review included studies published before 9 April 2018. Accordingly, for studies published before 9 April 2018, all the articles identified by He et al. were considered, and those meeting the inclusion criteria of the present analysis (reporting an externally validated score for predicting long-term outcomes after liver resection for colorectal metastases) were retained for analysis. For the period between 9 April 2018 and 1 June 2024, an independent literature search was conducted, and all the identified studies were screened.

### 2.3. Study Selection

The extracted papers were screened using the Rayyan platform (https://www.rayyan.ai/ (accessed on 1 June 2024) [20]. Duplicate articles were automatically detected by the software, manually verified, and excluded if confirmed as duplicates by one author (LV). No additional automated tools were employed. The titles and abstracts of the remaining articles were independently assessed by two authors (ED and LR), with any conflicts resolved by a third independent author (LV). The full texts of the selected articles were then retrieved and thoroughly reviewed by three authors (LV, ED, and LR) to confirm their eligibility according to the inclusion and exclusion criteria. The reference list of all the included articles was then screened for additional articles.

### 2.4. Data Extraction

For each article included in the study, the following data were extracted: (1) article metrics, including first author, institution, year of publication, journal, study design, number of institutions involved, and enrollment period; (2) patient characteristics of the validation cohort including number of individuals, age, sex, primary tumor site, preoperative chemotherapy, number and size of liver metastases, synchronous presentation, and long-term outcomes; (3) externally validated prognostic scores including number and type of scores, variables used to define the score, number of classes, predicted outcome, type of metric used for score evaluation, and statistical performance.

Three authors (LV, ED, and LR) independently extracted all data from the selected papers, with at least two authors reviewing each paper. Data were cross-checked by the two authors, and any discrepancies between reviewers were resolved through consensus discussions. When data were presented only in graphs or images and not detailed in the text, values were extracted using the DigitizeIt software version 2.5.10 (Bormisoft, Braunschweig, Germany—available at https://www.digitizeit.xyz/it/ (accessed on 1 June 2024)).

### 2.5. Quality Assessment

The Prediction Model Risk of Bias Assessment Tool (PROBAST) [21] was used to evaluate the quality of the included studies in terms of risk of bias and applicability. In every single study, the PROBAST assessment was performed for each model that met the inclusion criteria for the systematic review. The risk of bias was evaluated across four domains (participants, predictors, outcome, and analysis), while applicability was assessed across three domains (participants, predictors, and outcome). Quality appraisal was conducted independently by two authors (LV and ER). In cases of disagreement, a third author (LR) was consulted to reach a consensus.

### 2.6. Statistical Analysis

Categorical variables were presented as numbers and percentages. Continuous variables were reported as mean ± standard deviation or median with interquartile range. For homogeneous reporting of patient characteristics, whenever articles reported continuous data as median and range (or interquartile range), the mean and standard deviation were estimated using the formulas proposed by Luo et al. and Wan et al. [22,23]. Comparisons between categorical variables were performed using the chi-square test or Fisher’s exact test, as appropriate. The Shapiro–Wilk test was used to assess the normality of continuous variable distributions. Depending on the distribution, continuous variables were compared using either the unpaired *t*-test (for normally distributed data) or the Mann–Whitney U test (for non-normally distributed data).

A meta-analysis of performance was conducted for each prognostic model validated by at least two articles and with performance expressed as C-index (according to Harrell’s version) [24] or AUC (area under the ROC curve) statistics [25]. Whenever the standard error (SE) of the performance indices was missing, it was estimated using the formula provided by Altman et al. [26] if confidence intervals were available, and the method proposed by Debray et al. [27] otherwise. Whenever a score was validated using multiple metrics or for multiple outcomes, separate meta-analyses were conducted for each; non-time-dependent metrics (C-index or AUC) and time-dependent ones (1-, 3-, and 5-year AUCs or C-indices) were the object of separate meta-analyses. Given the small number of included studies for each score and the heterogeneity across extracted data and clinical settings, a random-effects model using restricted maximum likelihood (REML) estimation was adopted to pool results along with the Hartung–Knapp–Sidik–Jonkman (HKSJ) method to estimate 95% confidence interval (95%CI) [28]. The Cochrane Q test and the I2 statistic were used to evaluate statistical heterogeneity. I2 cut-off values of 25%, 50%, and 75% were adopted to indicate low, moderate, and high heterogeneity, respectively [29]. When performance values for the same population and score were available at different time points (e.g., 1-, 3-, and 5-year C-indices/AUCs), a meta-regression was performed to assess whether performance varied across time points. Finally, whenever a score was analyzed using a different number of classes (e.g., the Fong score analyzed for five separate classes or grouped as 0–2 vs. 3–5), a meta-regression was performed to evaluate the impact of the number of classes on performance. The 95% prediction interval (PI) was calculated by incorporating heterogeneity into the summarized C-statistics to define a range of C-statistics values within which future validations should fall. Funnel plots were visually evaluated to check for any publication bias; the Egger regression-based test was then applied to assess for small-study effect [30]. Whenever the Egger test was significant, the trim-and-fill method was applied to assess the impact of the publication bias on the results. *p*-values lower than 0.05 were considered significant for all the analyses. The C-statistic value was considered significant if the 95%CI excluded the 0.5 value. According to Hosmer and Lemeshow [31], the discrimination capability of the models was classified as follows: no discrimination if AUC ≤ 0.500; poor if AUC 0.501–0.700; good if 0.701–0.800; excellent if 0.801–0.900; and outstanding if >0.900. Statistical analysis was performed using STATA/SE version 18 (StataCorp, College Station, TX, USA).

## 3. Results

**Figure 1:**
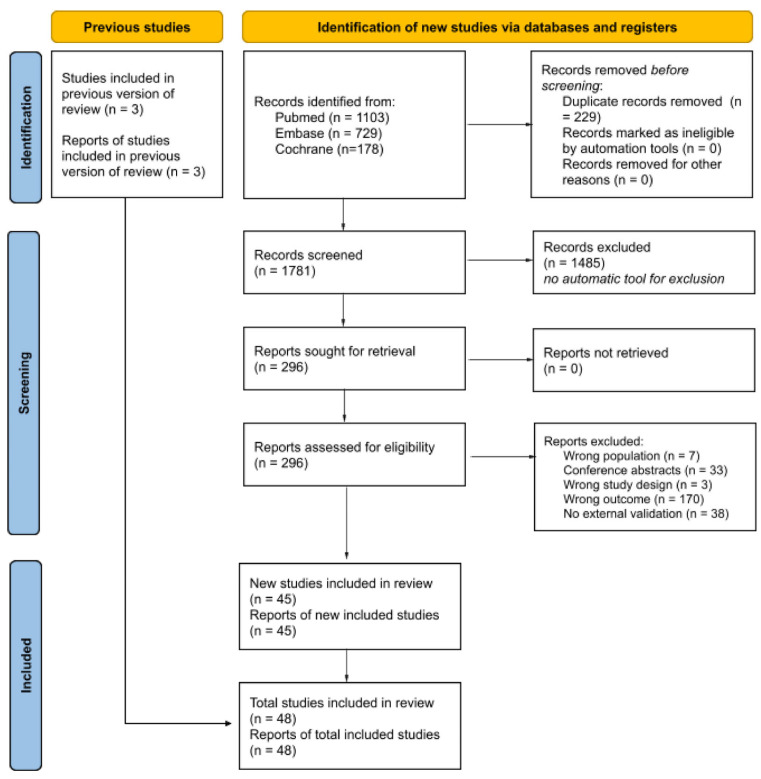
PRISMA 2020 flowchart representing the selection process.

The online search conducted on the PubMed, Embase, and Cochrane databases identified 2010 papers published after 9 April 2018. After the exclusion of duplicates, 1781 articles were screened, and 1485 were excluded based on their titles and abstracts. The full text of the remaining 296 papers was retrieved and analyzed. Forty-five articles were retained for the present analysis. Three additional articles were selected from the review by He et al. (up to 9 April 2018) [19], resulting in a total of 48 included studies [16,17,32,33,34,35,36,37,38,39,40,41,42,43,44,45,46,47,48,49,50,51,52,53,54,55,56,57,58,59,60,61,62,63,64,65,66,67,68,69,70,71,72,73,74,75,76,77]. The selection process (PRISMA flowchart) is summarized in Figure 1.

All the studies were retrospective, with 32 (67%) being multicentric. Most papers were published in the last five years (40 out of 48, 83%, since 2020). Of the 48 studies, 22 involved Chinese centers, 18 European centers, and 13 U.S. centers. Table 1 summarizes the study details.

Overall, 33,602 patients were analyzed, resulting from 69 cohorts of patients in the 48 papers (median number of patients per cohort 341, IQR 174–672, range 28–4112). The patients’ characteristics are summarized in the Supplementary Table S2.

Thirteen studies included patients treated with thermal ablation, representing less than 15% of each series. In all but one study [72], ablation was performed intraoperatively and in combination with liver resection.

The studies validated 48 scores, with the Fong score being the most frequently validated (32 studies), followed by the GAME score (11 studies), and the RASmut-CRS (8 studies). Of the analyzed scores, 40 (83%) were based on preoperative data and 8 (17%) on pre- and postoperative data. Seventeen (35%) scores were based on standard clinical and laboratory data, while 31 (65%) included additional variables (in 24 cases combined with clinical data): 17 (35%) included genetic data, 8 (17%) included inflammatory/immunological data, and 6 (13%) included advanced imaging analysis data.

**Figure 2:**
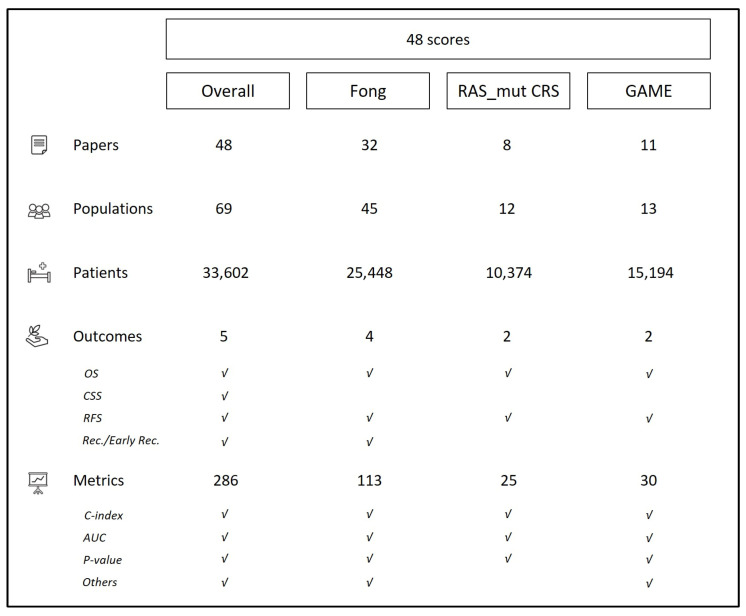
Summary of analyzed outcomes and metrics for most frequently validated prognostic scores.

Nine (19%) scores have been validated by more than one paper, and 12 (25%) in more than one cohort of patients. The authors analyzed five different outcomes: overall survival, recurrence-free survival, cancer-specific survival, recurrence rate, and early recurrence rate (recurrence within 6 months after surgery). The most commonly adopted metrics were the C-index (for 34 scores), AUC (for 19), and *p*-value (for 11). Figure 2 summarizes the analyzed outcomes and adopted metrics.

Regarding quality assessment, 28 (58%) of the included studies were found to have a high risk of bias, and 13 (27%) raised concerns about applicability. The high risk of bias was primarily due to inadequate methodology for external validation or incomplete reporting (26 studies, 54% of the total). Overall, only 15 (31%) studies were rated as having both a low risk of bias and no concerns about applicability. Detailed data about quality assessment are reported in Supplementary Table S3.

### 3.1. Overall Survival

The detailed performance of the scores assessed for overall survival prediction is reported in Supplementary Table S4.

**Figure 3:**
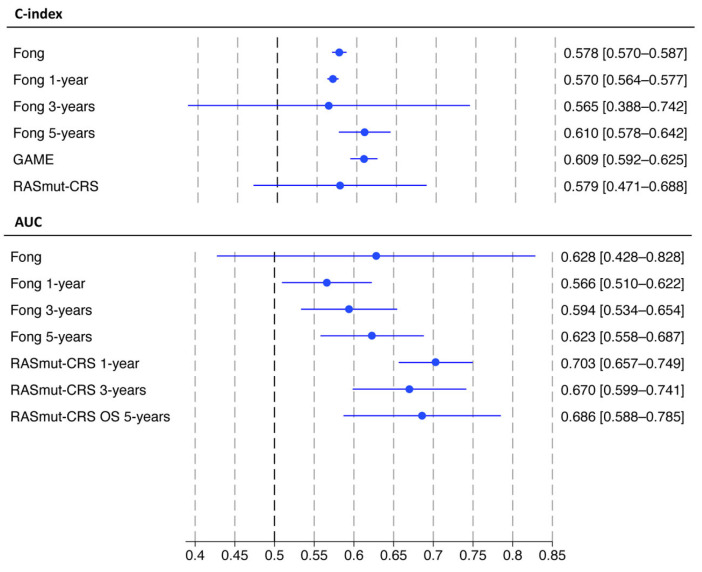
Meta-analysis for each score predicting overall survival.

Thirteen meta-analyses were performed for three scores. The pooled C-index was 0.609 (95%CI 0.592–0.625) for the GAME score, and 0.578 (95%CI 0.570–0.587) for the Fong score. The RASmut-CRS had a pooled C-index of 0.579 with 95%CI crossing the 0.5 value (0.471–0.688). The pooled AUC was calculated only for the Fong score and was 0.628 with 95%CI crossing the 0.5 value (0.428–0.828). Focusing on time-dependent metrics, the pooled 1-, 3- and 5-year C-indices were available only for the Fong score: 0.570 (95%CI 0.564–0.577), 0.565 (95%CI 0.388–0.742) and 0.610 (95%CI 0.578–0.642), respectively. The 1-, 3- and 5-year AUCs were available for the Fong score [0.566 (95%IC 0.510–0.622), 0.594 (95%CI 0.534–0.654), and 0.623 (95%CI 0.558–0.687), respectively], and for the RASmut-CRS [0.703 (95%CI 0.657–0.749), 0.670 (95%CI 0.599–0.741), and 0.686 (95%IC 0.588–0.785), respectively]. The 95%PI confirmed adequate performance for the Fong and GAME score. The results are summarized in Figure 3 and Table 2.

The meta-regression of time-dependent metrics, with follow-up time as predictor, showed a significant increase in Fong score performance through the years: 0.01 increase per year of the pooled C-index, *p* = 0.039. The meta-regression, with the number of classes as a predictor, showed no significant differences.

### 3.2. Recurrence-Free Survival

The detailed performance of the scores assessed for recurrence-free survival prediction is reported in Supplementary Table S5.

**Figure 4:**
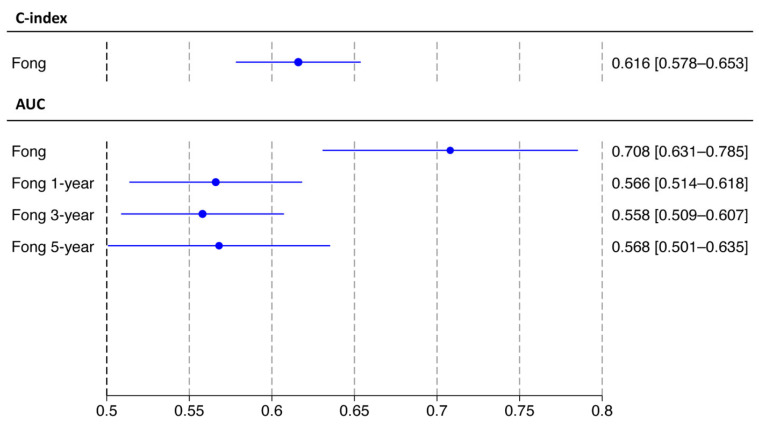
Meta-analysis for each score predicting recurrence-free survival.

Five meta-analyses were performed only for the Fong score. The pooled C-index was 0.616 (95%CI 0.578–0.653), and the pooled AUC was 0.708 (95%CI 0.631–0.785). Focusing on time-dependent metrics, the 1-, 3- and 5-year pooled AUCs were 0.566 (95%CI 0.514–0.618), 0.558 (95%CI 0.509–0.607) and 0.568 (95%CI 0.501–0.635), respectively. The 95%PI confirmed adequate performance for the Fong score. The results are summarized in Figure 4 and Table 2.

### 3.3. Other Outcomes

Three additional outcomes have been analyzed. Guo et al. developed and validated, within the same study, a score for predicting cancer-specific survival, reporting a C-index of 0.67 [63]. Considering the recurrence rate, Wada et al. externally validated two scores based on transcriptomic data and transcriptomic data combined with clinical ones and reported an AUC of 0.81 (95%CI 0.74–0.87) and 0.85 (95%CI 0.78–0.90), respectively [61]. Considering the early recurrence risk (≤6 months after surgery), Dai et al. evaluated the Fong and Beppu scores, reporting AUCs of 0.654 and 0.686, respectively [77].

### 3.4. Variables Included in the Models

**Figure 5:**
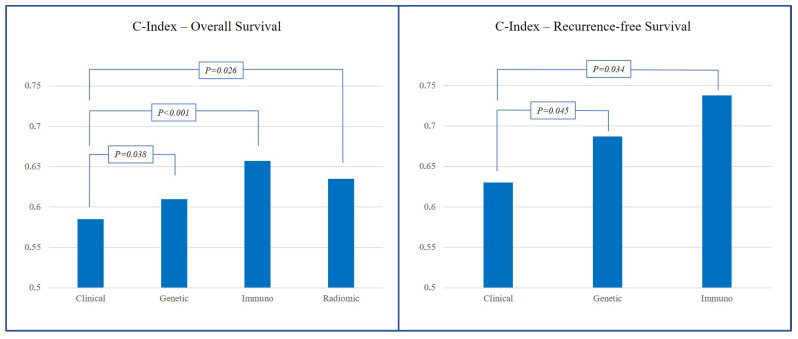
Comparison of performance between scores grouped by the type of predictors.

The median performance of the scores based on the preoperative data was lower than that of the scores based on the pre- and postoperative data (for OS, median C-index 0.600 vs. 0.671, *p* = 0.007). The scores based solely on clinical data had the lowest performance (median C-index for OS 0.585 and for RFS 0.630), lower than the studies including genetic data (C-index for OS 0.610, *p* = 0.038; C-index for RFS 0.687, *p* = 0.045), inflammatory/immunological data (C-index for OS 0.657, *p* < 0.001; C-index for RFS 0.738, *p* = 0.034, respectively), or imaging-based data (C-index for OS 0.635, *p* = 0.026, not available for RFS). Data are summarized in Figure 5.

### 3.5. Publication Bias

Funnel plots constructed for each score are reported in Supplementary Figure S1. The Egger test showed a possible small-study effect only for the “GAME OS C-index” meta-analysis. A trim-and-fill method was applied, finding only two imputed studies and a small decrease in the pooled result (0.605, 95%CI 0.592–0.619 vs. 0.609, 95%CI 0.592–0.625). This suggested that publication bias was not substantial and unlikely to impact any conclusions.

## 4. Discussion

The present meta-analysis highlights a strong research focus on prognostic scores for patients with CRLM, with 48 different models having undergone external validation. Nevertheless, three critical limitations emerged: standard clinical prognostic scores show low performance and poor discriminative ability; the reporting of results is highly heterogeneous across studies, limiting the feasibility of comprehensive meta-analysis; and innovative prognostic scores are hindered by limited validation and concerns regarding reproducibility.

Although the clinical usefulness of prognostic scores is debated [78], clinicians continue to require reliable tools to support decision-making. Modern surgeons must face the concept of ‘oncological resectability’, weighing indications according to survival benefits after treatment rather than merely based on the technical feasibility of surgery [11,79,80,81]. Therapeutic alternatives to surgery—thermal ablation for oligometastatic disease and liver transplantation for diffuse tumors [13,14]—can be considered, with the choice depending on the patient’s prognosis and the expected treatment outcomes. In patients with CRLM, prognostic scores are widely used in both daily clinical practice and research settings [82,83,84,85,86]. For example, the CHARISMA trial [82] selects patients for preoperative chemotherapy based on their Fong score. The present meta-analysis raises an important concern: the Fong score demonstrated poor performance in predicting overall survival (C-index = 0.58; AUC = 0.63), misclassifying more than one-third of patients. This limited prognostic accuracy may be attributed to major advances in CRLM management since the score was introduced in 1999, including the widespread adoption of preoperative systemic therapies, improvements in staging and patient selection, and increasingly aggressive surgical indications [7,9,79,87,88,89]. However, newer prognostic scores developed to better reflect contemporary CRLM management and incorporate genetic data, namely the GAME score proposed in 2018 [17] and the RASmut-CRS introduced in 2019 [16], failed to substantially improve prognostic performance. In the present meta-analysis, the prediction of overall survival remained consistently poor (C-index = 0.58 for RASmut-CRS and 0.61 for the GAME score), with only a modest improvement observed for recurrence-free survival (C-index = 0.687 for RASmut-CRS).

Should prognostic scores incorporate new data beyond clinical variables? Two main research areas have been actively explored in recent years. On the one hand, growing evidence supports the prognostic impact of immunological and inflammatory factors, including both laboratory-based indices (e.g., lymphocyte-to-neutrophil ratio and C-reactive protein) and pathological features, such as immune infiltrates within the tumor and peritumoral tissue [46,53,58,71,90]. On the other hand, radiomics has gained traction due to its ability to extract quantitative indices from medical imaging that correlate with pathological characteristics and survival outcomes [91,92], including in patients with CRLM [45,93]. In the present analysis, prognostic scores incorporating immunological or radiomic data demonstrated superior performance compared with purely clinical models, confirming their potential. However, these metrics could not be included in a formal meta-analysis, as most were validated only by the original reporting authors, and their inclusion in daily clinical practice has yet to be accomplished. An additional finding deserves attention: postoperative scores demonstrated better performance than preoperative ones. This advantage underscores the prognostic relevance of intraoperative data (e.g., operative time and blood loss), postoperative variables (e.g., morbidity and administration of adjuvant chemotherapy), and pathological findings (e.g., surgical margins, microvascular invasion, and pathological response to chemotherapy) [88,94,95,96,97,98], all of which present a major challenge for the development of purely preoperative prognostic scoring systems.

The present analysis also highlights some relevant methodological considerations. Despite the large volume of available data (69 patient cohorts, 33,602 patients, and 286 performance metrics), the meta-analysis could be conducted on only a small subset of prognostic models (3 of 48 scores). This limitation was primarily driven by heterogeneity and inadequacies in statistical analyses and reporting, as evidenced by the high risk of bias identified in 58% of the studies. Statistical analyses were inadequate in approximately 10% of the studies, relying on visual comparisons of survival curves or *p*-values, while the remaining studies used a wide range of performance measures (C-index, AUC, AIC, and K-index), including time-dependent values assessed at fixed points ranging from one to ten years after surgery. Reporting quality further limited data synthesis, as some studies presented results only graphically without providing detailed numerical values in the text or confidence intervals. Because meta-analyses require homogeneous, complete, and comparable data, a substantial proportion of studies and performance metrics had to be excluded. Finally, the AUC estimates were consistently higher than the corresponding C-index values, raising concerns about potential overfitting and overestimation of model performance.

Some limitations of the present study should be acknowledged. All the included analyses were retrospective and exhibited substantial heterogeneity regarding patient selection criteria and reported outcome measures. Only models that had been externally validated by multiple studies were considered, thereby excluding potentially emerging approaches. Finally, the meta-analysis was based on a relatively small number of studies, with considerable statistical heterogeneity in some cases, which may introduce a risk of bias. Nevertheless, the present study is clinically relevant for at least three reasons: First, it reveals the disappointing performance of currently used prognostic scores—major clinical decisions are based on predictions that fail in more than one-third of patients, a fact that clinicians must be aware of. Second, promising results are emerging from radiomic and immuno-related scores [99,100,101]. This should guide future research and efforts. Finally, we highlighted the urgent need for standardization in both analyses and reporting. Guidelines and checklists should clearly specify the approaches to be pursued and the outcome measures to be detailed, and methodological supervision by a statistician should be mandatory.

## 5. Conclusions

In conclusion, the currently used prognostic scores have inadequate performance, with inaccurate predictions in more than one-third of patients. New approaches incorporating novel prognostic factors are needed, but clear standards for statistical methodology and reporting should be established to ensure the generation of robust and reliable evidence.

## Figures and Tables

**Table 1:** Study details.

Author	Year	Country	Study Design		Period (Validation)	Patients (Validation)	Outcome	Selection Criteria
Skipenko [32]	2015	Russia	R	Mono	1991–2014	312	OS	/
Sasaki [33]	2018	US, Japan, Italy	R	Multi	2000–2015	430/198	OS	/
Wang [34]	2018	China	R	Mono	2002–2015	249	RFS	No EH
Margonis [17]	2018	US	R	Multi	2000–2015	747/502	OS	/
Liu W [70]	2019	China	R	Multi	2013–2017	117	RFS	Preoperative CTx
Duprè [71]	2019	US, China, France	R	Multi	2010–2015	219	OS	/
Brudvik [16]	2019	US, EU, Japan	R	Multi	2005–2013	608/564	OS, RFS	/
Gasser [76]	2019	Japan, EU	R	Multi	2005–2016	527	OS, RFS	/
Chen Y [68]	2020	China	R	Mono	2010–2018	787/162	RFS	/
Kim WJ [69]	2020	Korea	R	Mono	2002–2015	295	RFS	No EH, No non-cancer-related death
Paredes [75]	2020	US, EU	R	Multi	2001–2018	703/703	RFS	/
Bao X [49]	2021	China, US	R	Multi	8 years	312/144	OS	/
Fruhling [62]	2021	Sweden	R	Multi	2005–2015	1212	OS	/
Guo X [63]	2021	US, China	R	Multi	2010–2017	112	OS, DSS	Synchronous CRLM, Simultaneous res.
Liu W [64]	2021	China	R	Multi	2009–2018	237/532	RFS	No EH
Takeda [65]	2021	Japan	R	Multi	2010–2016	309	OS	/
Kawaguchi Y [66]	2021	US, Japan, Italy	R	Multi	2006–2018	254/419	OS	/
Sasaki [67]	2021	US, Argentina, EU, Japan	R	Multi	2000–2018	2376	OS	/
Bai L [73]	2021	China	R	Multi	2000–2019	580	OS	/
Meng Q [74]	2021	China	R	Mono	2010–2019	174/60	OS	Synchronous CRLM, Simultaneous res. No EH, Adjuvant CTx
Dai S [77]	2021	China	R	Mono	2012–2019	202	Early rec	/
Chen Q [48]	2022	China	R	Mono	2012–2018	389	OS, RFS	/
Chen FL [50]	2022	China	R	Mono	2000–2020	375 + 424 + 296	OS	No EH
Villard [51]	2022	EU	R	Multi	2007–2018	391/1013	OS	/
Zhou Z [52]	2022	China	R	Mono	2009–2019	118	RFS	No EH, Preoperative CTx
Filippini Velazquez [53]	2022	Germany	R	Multi	2006–2016	230	OS	/
Buisman [54]	2022	The Neth., US	R	Multi	1992–2019	3064/1048/4112	OS	/
Paro [55]	2022	US, EU	R	Multi	2001–2018	672	OS	/
Bai L [56]	2022	China	R	Multi	2001–2016	325/341	OS, RFS	R0 resection
Sasaki [57]	2022	US, Korea, Japan, EU	R	Multi	2004–2019	1307/1058 and 2365/1205	OS	/
Furukawa [58]	2022	Japan	R	Mono	2002–2018	149	OS, RFS	/
Wong GYM [59]	2022	Australia	R	Mono	2007–2017	103	OS, RFS	/
Zhai Y [60]	2022	China	R	Mono	2010–2016	147	RFS	No preoperative CTx
Wada Y [61]	2022	Japan	R	Multi	2001–2016	151	OS, RFS, Recurrence	/
Bolhuis [72]	2022	The Neth.	R	Multi	2015–2016	1105	OS, RFS	No EH
Qi [40]	2023	China	R	Multi	2010–2019	433/404	OS	No EH
Lam [41]	2023	China	R	Multi	2009–2018	172	OS	/
Katipally [42]	2023	US, UK	R	Multi	2007–2012	147	OS, RFS	No EH, Perioperative CTx
Li [43]	2023	China	R	Multi	2012–2020	122	OS	Synchronous CRLM, No EH, Young onset
Reijonen [44]	2023	Finland	R	Mono	2000–2019	816	OS	/
Chen J [45]	2023	Canada	R	Multi	2006–2012	28	OS	Preoperative CTx
Zhang C [46]	2023	China	R	Multi	2014–2019	106/95	OS, RFS	/
Beppu [47]	2023	Japan	R	Multi	2005–2007	1756/469	OS, RFS	/
Ding [35]	2024	US, China	R	Multi	2010–2022	102	OS	Synchronous CRLM, Preoperative CTx
Chen [36]	2024	China	R	Multi	NA	230	OS, RFS	Synchronous CRLM, Simultaneous res.
Jiang [37]	2024	China	R	Mono	2009–2019	371	OS	No EH
Martin-Cullell [38]	2024	Spain	R	Mono	2004–2020	176	OS, RFS	/
Takematsu [39]	2024	Japan	R	Mono	2004–2020	218	OS, RFS	/

R: retrospective; Mono: monocentric study; Multi: multicentric study, DSS: disease-specific survival; RFS: recurrence-free survival; OS: overall survival; CTx: chemotherapy; EH: extrahepatic disease; res: resection.

**Table 2:** Meta-analysis of performances of each score for overall and recurrence-free survival.

Score	Metric	Articles	Populations	Patients	Pooled Result	CI 95%	I2	PI 95%
**Overall Survival**								
**C-index**								
Fong	C-index	8	9	6376	0.578	0.570–0.587	<0.01%	0.570–0.587
	1-year C-index	2	2	1277	0.570	0.564–0.577	<0.01%	-
	3-year C-index	2	2	1417	0.565	0.388–0.742	47.67%	-
	5-year C-index	5	6	2835	0.610	0.578–0.642	64.05%	0.536–0.684
GAME	C-index	5	6	7078	0.609	0.592–0.625	0.00%	0.591–0.627
RASmut-CRS	C-index	3	3	2850	0.579	0.471–0.688	79.59%	-
**AUC**								
Fong	AUC	2	2	4361	0.628	0.428–0.828	17.19%	-
	1-year AUC	4	6	1999	0.566	0.510–0.622	13.53%	0.483–0.648
	3-year AUC	4	6	1999	0.594	0.534–0.654	65.75%	0.454–0.735
	5-year AUC	4	6	2288	0.623	0.558–0.687	74.91%	0.457–0.788
RASmut-CRS	1-year AUC	2	4	1466	0.703	0.657–0.749	0.02%	0.640–0.765
	3-year AUC	2	4	1466	0.670	0.599–0.741	58.59%	0.494–0.845
	5-year AUC	2	4	1466	0.686	0.588–0.785	78.09%	0.417–0.956
**Recurrence-free Survival**								
**C-index**								
Fong	C-index	4	5	2434	0.616	0.578–0.653	10.9%	0.561–0.671
**AUC**								
Fong	AUC	3	3	662	0.708	0.631–0.785	0.02%	0.480–0.936
	1-year AUC	4	8	4387	0.566	0.514–0.618	97.54%	0.424–0.708
	3-year AUC	3	6	3438	0.558	0.509–0.607	97.07%	0.439–0.677
	5-year AUC	3	6	3438	0.568	0.501–0.635	98.80%	0.389–0.748

## Data Availability

Data are available upon reasonable request.

## Supplementary Materials

The following supporting information can be downloaded at https://www.mdpi.com/article/10.3390/cancers18040625/s1. Supplementary Figure S1: Funnelplots for each score included in the meta-analysis; Supplementary Table S1: PRISMA 2020 checklist; Supplementary Table S2: Patient characteristics; Supplementary Table S3: Quality assessment according to the Prediction Model Risk of Bias Assessment Tool (PROBAST); Supplementary Table S4: Performances of the score for OS prediction; Supplementary Table S5: Performances of the score for RFS prediction.

## Abbreviations

The following abbreviations are used in this manuscript:

CRLM	Colorectal liver metastases
RAS-mut CRS	RAS mutation Clinical Risk Score
OS	Overall survival
RFS	Regression-free survival
SE	Standard error
REML	Restricted maximum likelihood
HKSJ	Hartung–Knapp–Sidik–Jonkman
95%CI	95% confidence interval
95%PI	95% prediction interval
IQR	Interquartile range

## References

[1] Morgan E., Arnold M., Gini A., Lorenzoni V., Cabasag C.J., Laversanne M., Vignat J., Ferlay J., Murphy N., Bray F. (2023). Global burden of colorectal cancer in 2020 and 2040: Incidence and mortality estimates from GLOBOCAN. Gut.

[2] Bray F., Laversanne M., Sung H., Ferlay J., Siegel R.L., Soerjomataram I., Jemal A. (2024). Global cancer statistics 2022: GLOBOCAN estimates of incidence and mortality worldwide for 36 cancers in 185 countries. CA Cancer J. Clin..

[3] Cervantes A., Adam R., Roselló S., Arnold D., Normanno N., Taïeb J., Seligmann J., De Baere T., Osterlund P., Yoshino T. (2023). Metastatic colorectal cancer: ESMO Clinical Practice Guideline for diagnosis, treatment and follow-up. Ann. Oncol..

[4] Viganò L., Jayakody Arachchige V.S., Fiz F. (2022). Is precision medicine for colorectal liver metastases still a utopia? New perspectives by modern biomarkers, radiomics, and artificial intelligence. World J. Gastroenterol..

[5] Adam R., de Gramont A., Figueras J., Kokudo N., Kunstlinger F., Loyer E., Poston G., Rougier P., Rubbia-Brandt L., Sobrero A. (2015). Managing synchronous liver metastases from colorectal cancer: A multidisciplinary international consensus. Cancer Treat. Rev..

[6] Kawaguchi Y., Kopetz S., Panettieri E., Hwang H., Wang X., Cao H.S.T., Tzeng C.D., Chun Y.S., Aloia T.A., Vauthey J.N. (2022). Improved Survival over Time After Resection of Colorectal Liver Metastases and Clinical Impact of Multigene Alteration Testing in Patients with Metastatic Colorectal Cancer. J. Gastrointest. Surg..

[7] Adam R., Pascal G., Castaing D., Azoulay D., Delvart V., Paule B., Levi F., Bismuth H. (2004). Tumor progression while on chemotherapy: A contraindication to liver resection for multiple colorectal metastases?. Ann. Surg..

[8] Viganò L., Terrone A., Costa G., Franchi E., Cimino M., Procopio F., Del Fabbro D., Torzilli G. (2022). Effect of chemotherapy on tumour-vessel relationship in colorectal liver metastases. Br. J. Surg..

[9] Mason M.C., Krasnodebski M., Hester C.A., Kothari A.N., Barker C., Nishioka Y., Chiang Y.J., Newhook T.E., Tzeng C.D., Chun Y.S. (2022). Outcomes of Mixed Pathologic Response in Patients with Multiple Colorectal Liver Metastases Treated with Neoadjuvant Chemotherapy and Liver Resection. Ann. Surg. Oncol..

[10] Tian Y., Wang Y., Wen N., Wang S., Li B., Liu G. (2024). Prognostic factors associated with early recurrence following liver resection for colorectal liver metastases: A systematic review and meta-analysis. BMC Cancer.

[11] Viganò L., Gentile D., Galvanin J., Corleone P., Costa G., Cimino M., Procopio F., Torzilli G. (2022). Very early recurrence after liver resection for colorectal metastases: Incidence, risk factors, and prognostic impact. J. Gastrointest. Surg..

[12] Hirokawa F., Ueno M., Nakai T., Kaibori M., Nomi T., Iida H., Tanaka S., Komeda K., Hayami S., Kosaka H. (2022). Neoadjuvant Chemotherapy Versus Upfront Surgery for Resectable Liver Metastases from Colorectal Cancer: A Multicenter, Propensity Score-Matched Cohort Study. J. Gastrointest. Surg..

[13] Bonney G.K., Chew C.A., Lodge P., Hubbard J., Halazun K.J., Trunecka P., Muiesan P., Mirza D.F., Isaac J., Laing R.W. (2021). Liver transplantation for non-resectable colorectal liver metastases: The International Hepato-Pancreato-Biliary Association consensus guidelines. Lancet Gastroenterol. Hepatol..

[14] van der Lei S., Puijk R.S., Dijkstra M., Schulz H.H., Vos D.J.W., De Vries J.J.J., Scheffer H.J., Lissenberg-Witte B.I., Aldrighetti L., Arntz M. (2025). Thermal ablation versus surgical resection of small-size colorectal liver metastases (COLLISION): An international, randomised, controlled, phase 3 non-inferiority trial. Lancet Oncol..

[15] Fong Y., Fortner J., Sun R.L., Brennan M.F., Blumgart L.H. (1999). Clinical score for predicting recurrence after hepatic resection for metastatic colorectal cancer: Analysis of 1001 consecutive cases. Ann. Surg..

[16] Brudvik K.W., Jones R.P., Giuliante F., Shindoh J., Passot G., Chung M.H., Song J., Li L., Dagenborg V.J., Fretland Å.A. (2019). RAS mutation clinical risk score to predict survival after resection of colorectal liver metastases. Ann. Surg..

[17] Margonis G.A., Sasaki K., Gholami S., Kim Y., Andreatos N., Rezaee N., Deshwar A., Buettner S., Allen P.J., Kingham T.P. (2018). Genetic And Morphological Evaluation (GAME) score for patients with colorectal liver metastases. Br. J. Surg..

[18] Page M.J., McKenzie J.E., Bossuyt P.M., Boutron I., Hoffmann T.C., Mulrow C.D., Shamseer L., Tetzlaff J.M., Akl E.A., Brennan S.E. (2021). The PRISMA 2020 statement: An updated guideline for reporting systematic reviews. BMJ.

[19] He Y., Ong Y., Li X., Din F.V., Brown E., Timofeeva M., Wang Z., Farrington S.M., Campbell H., Dunlop M.G. (2019). Performance of prediction models on survival outcomes of colorectal cancer with surgical resection: A systematic review and meta-analysis. Surg. Oncol..

[20] Ouzzani M., Hammady H., Fedorowicz Z., Elmagarmid A. (2016). Rayyan-a web and mobile app for systematic reviews. Syst. Rev..

[21] Wolff R.F., Moons K.G.M., Riley R.D., Whiting P.F., Westwood M., Collins G.S., Reitsma J.B., Kleijnen J., Mallett S., PROBAST Group (2019). PROBAST: A tool to assess the risk of bias and applicability of prediction model studies. Ann. Intern. Med..

[22] Luo D., Wan X., Liu J., Tong T. (2018). Optimally estimating the sample mean from the sample size, median, mid-range, and/or mid-quartile range. Stat. Methods Med. Res..

[23] Wan X., Wang W., Liu J., Tong T. (2014). Estimating the sample mean and standard deviation from the sample size, median, range and/or interquartile range. BMC Med. Res. Methodol..

[24] Harrell F.E., Califf R.M., Pryor D.B., Lee K.L., Rosati R.A. (1982). Evaluating the yield of medical tests. JAMA.

[25] Guo C., So Y., Jang W. (2017). Evaluating Predictive Accuracy of Survival Models with PROC PHREG.

[26] Altman D.G., Bland J.M. (2005). Standard deviations and standard errors. BMJ.

[27] Debray T.P., Damen J.A., Snell K.I., Ensor J., Hooft L., Reitsma J.B., Riley R.D., Moons K.G. (2017). A guide to systematic review and meta-analysis of prediction model performance. BMJ.

[28] IntHout J., Ioannidis J.P.A., Borm G.F. (2014). The Hartung-Knapp-Sidik-Jonkman method for random effects meta-analysis is straightforward and considerably outperforms the standard DerSimonian-Laird method. BMC Med. Res. Methodol..

[29] Higgins J.P.T., Thompson S.G. (2002). Quantifying heterogeneity in a meta-analysis. Stat. Med..

[30] Egger M., Smith G.D., Phillips A.N. (1997). Meta-analysis: Principles and procedures. BMJ.

[31] Hosmer D.W., Lemeshow S., Sturdivant R.X. (2013). Applied Logistic Regression.

[32] Skipenko O.G., Bedzhanyan A.L., Polishchuk L.O. (2015). The role of prognostic models in metastatic colorectal liver cancer surgery. Khirurgiia.

[33] Sasaki K., Morioka D., Conci S., Margonis G.A., Sawada Y., Ruzzenente A., Kumamoto T., Iacono C., Andreatos N., Guglielmi A. (2018). The Tumor Burden Score: A New “Metro-ticket” Prognostic Tool For Colorectal Liver Metastases Based on Tumor Size and Number of Tumors. Ann. Surg..

[34] Wang Y., Lin H.C., Huang M.Y., Shao Q., Wang Z.Q., Wang F.H., Yuan Y.F., Li B.K., Wang D.S., Ding P.R. (2018). The Immunoscore system predicts prognosis after liver metastasectomy in colorectal cancer liver metastases. Cancer Immunol. Immunother..

[35] Ding Y., Han X., Zhao S., Wang S., Guo J., Leng C., Li X., Wang K., Qiu W., Qi W. (2024). Constructing a prognostic model for colorectal cancer with synchronous liver metastases after preoperative chemotherapy: A study based on SEER and an external validation cohort. Clin. Transl. Oncol..

[36] Chen Q., Chen J., Deng Y., Bi X., Zhao J., Zhou J., Huang Z., Cai J., Xing B., Li Y. (2024). Personalized prediction of postoperative complication and survival among Colorectal Liver Metastases Patients Receiving Simultaneous Resection using machine learning approaches: A multi-center study. Cancer Lett..

[37] Jiang C., Liu W., Jin Z., Lan L., Xu L., Du A., Peng S., Zeng Y., Wang H., Liao M. (2024). Construction and validation of “WCH-nomogram” for predicting the prognosis after resection of colorectal liver metastases. Cancer Med..

[38] Martín-Cullell B., Virgili A.C., Riera P., Fumagalli C., Mirallas O., Pelegrín F.J., Sánchez-Cabús S., Molina V., Szafranska J., Páez D. (2024). Histopathological, Clinical, And Molecular (HICAM) score for patients with colorectal liver metastases. Br. J. Surg..

[39] Takematsu T., Mima K., Hayashi H., Kitano Y., Nakagawa S., Hiyoshi Y., Okabe H., Imai K., Miyamoto Y., Baba H. (2024). RAS mutation status in combination with the JSHBPS nomogram may be useful for preoperative identification of colorectal liver metastases with high risk of recurrence and mortality after hepatectomy. J. Hepatobiliary Pancreat. Sci..

[40] Qi L., Liang J.Y., Li Z.W., Xi S.Y., Lai Y.N., Gao F., Zhang X.R., Wang D.S., Hu M.T., Cao Y. (2023). Deep learning-derived spatial organization features on histology images predicts prognosis in colorectal liver metastasis patients after hepatectomy. iScience.

[41] Lam C.S.N., Bharwani A.A., Chan E.H.Y., Chan V.H.Y., Au H.L.H., Ho M.K., Rashed S., Kwong B.M.H., Fang W., Ma K.W. (2023). A machine learning model for colorectal liver metastasis post-hepatectomy prognostications. Hepatobiliary Surg. Nutr..

[42] Katipally R.R., Martinez C.A., Pugh S.A., Bridgewater J.A., Primrose J.N., Domingo E., Maughan T.S., Talamonti M.S., Posner M.C., Weichselbaum R.R. (2023). Integrated Clinical-Molecular Classification of Colorectal Liver Metastases: A Biomarker Analysis of the Phase 3 New EPOC Randomized Clinical Trial. JAMA Oncol..

[43] Li T., Liang Y., Wang D., Zhou Z., Shi H., Li M., Liao H., Li T., Lei X. (2023). Development and validation of a clinical survival model for young-onset colorectal cancer with synchronous liver-only metastases: A SEER population-based study and external validation. Front. Oncol..

[44] Reijonen P., Nordin A., Savikko J., Poussa T., Arola J., Isoniemi H. (2023). Histopathological Helsinki score of colorectal liver metastases predicts survival after liver resection. APMIS.

[45] Chen J., Cheung H.M.C., Karanicolas P.J., Coburn N.G., Martel G., Lee A., Patel C., Milot L., Martel A.L. (2023). A radiomic biomarker for prognosis of resected colorectal cancer liver metastases generalizes across MRI contrast agents. Front. Oncol..

[46] Zhang C., Wang X.Y., Zuo J.L., Wang X.F., Feng X.W., Zhang B., Li Y.T., Yi C.H., Zhang P., Ma X.C. (2023). Localization and density of tertiary lymphoid structures associate with molecular subtype and clinical outcome in colorectal cancer liver metastases. J. Immunother. Cancer.

[47] Beppu T., Yamamura K., Sakamoto K., Honda G., Kobayashi S., Endo I., Hasegawa K., Kotake K., Itabashi M., Hashiguchi Y. (2023). Validation study of the JSHBPS nomogram for patients with colorectal liver metastases who underwent hepatic resection in the recent era—A nationwide survey in Japan. J. Hepatobiliary Pancreat. Sci..

[48] Chen Q., Li M., Chen J., Huang Z., Chen X., Zhao H., Cai J. (2022). AST·MLR index and operation injury condition are novel prognostic predictor for the prediction of survival in patients with colorectal cancer liver metastases undergoing surgical resection. BMC Cancer.

[49] Bao X., Wang K., Liu M., Li B., Wang H., Jin K., Yan X., Zhang H., Bao Q., Xu D. (2021). Characterization of genomic alterations in colorectal liver metastasis and their prognostic value. Front. Cell Dev. Biol..

[50] Chen F.L., Wang Y.Y., Liu W., Xing B.C. (2022). Prognostic factors in colorectal liver metastases patients with various tumor numbers treated by liver resection: A single-center, retrospective study. World J. Surg. Oncol..

[51] Villard C., Abdelrafee A., Habib M., Ndegwa N., Jorns C., Sparrelid E., Allard M.A., Adam R. (2022). Prediction of survival in patients with colorectal liver metastases- development and validation of a prognostic score model. Eur. J. Surg. Oncol..

[52] Zhou Z., Han X., Sun D., Liang Z., Wu W., Ju H. (2022). A comprehensive prognostic model for colorectal cancer liver metastasis recurrence after neoadjuvant chemotherapy. Front. Oncol..

[53] Filippini Velázquez G., Schiele S., Gerken M., Neumaier S., Hackl C., Mayr P., Klinkhammer-Schalke M., Illerhaus G., Schlitt H.J., Anthuber M. (2022). Predictive preoperative clinical score for patients with liver-only oligometastatic colorectal cancer. ESMO Open.

[54] Buisman F.E., Giardiello D., Kemeny N.E., Steyerberg E.W., Höppener D.J., Galjart B., Nierop P.M.H., Balachandran V.P., Cercek A., Drebin J.A. (2022). Predicting 10-year survival after resection of colorectal liver metastases; an international study including biomarkers and perioperative treatment. Eur. J. Cancer.

[55] Paro A., Hyer M.J., Tsilimigras D.I., Guglielmi A., Ruzzenente A., Alexandrescu S., Poultsides G., Aucejo F., Cloyd J.M., Pawlik T.M. (2022). Machine Learning Approach to Stratifying Prognosis Relative to Tumor Burden after Resection of Colorectal Liver Metastases: An International Cohort Analysis. J. Am. Coll. Surg..

[56] Bai L., Yan X.L., Lu Y.X., Meng Q., Rong Y.M., Ye L.F., Pan Z.Z., Xing B.C., Wang D.S. (2022). Circulating Lipid- and Inflammation-Based Risk (CLIR) Score: A Promising New Model for Predicting Outcomes in Complete Colorectal Liver Metastases Resection. Ann. Surg. Oncol..

[57] Sasaki K., Margonis G.A., Moro A., Wang J., Wagner D., Gagnière J., Shin J.K., D’Silva M., Sahara K., Miyata T. (2022). Nontumor related risk score: A new tool to improve prediction of prognosis after hepatectomy for colorectal liver metastases. Surgery.

[58] Furukawa K., Onda S., Yanagaki M., Taniai T., Hamura R., Haruki K., Shirai Y., Tsunematsu M., Sakamoto T., Ikegami T. (2022). Significance of intra/post-operative prognostic scoring system in hepatectomy for colorectal liver metastases. Ann. Gastroenterol. Surg..

[59] Wong G.Y.M., Bhimani N., Mol B., Diakos C., de Reuver P., Molloy M.P., Hugh T.J. (2022). Performance of prognostic models incorporating KRAS mutation status to predict survival after resection of colorectal liver metastases. HPB.

[60] Zhai Y., Bai W., Zhou J., Dong Q., Zhang J. (2022). Effect of tumour size ratio on liver recurrence-free survival of patients undergoing hepatic resection for colorectal liver metastases. BMC Cancer.

[61] Wada Y., Shimada M., Morine Y., Ikemoto T., Saito Y., Baba H., Mori M., Goel A. (2022). A transcriptomic signature that predicts cancer recurrence after hepatectomy in patients with colorectal liver metastases. Eur. J. Cancer.

[62] Frühling P., Urdzik J., Strömberg C., Isaksson B. (2021). Composite Score: Prognostic tool to predict survival in patients undergoing surgery for colorectal liver metastases. BJS Open.

[63] Guo X., Liu Y., Liu L.J., Li J., Zhao L., Jin X.R., Yan W., Lin B.Q., Shi S., Li Z.Y. (2021). Development and validation of survival nomograms in colorectal cancer patients with synchronous liver metastases underwent simultaneous surgical treatment of primary and metastatic lesions. Am. J. Cancer Res..

[64] Liu W., Zhang W., Xu Y., Li Y.H., Xing B.C. (2021). A Prognostic Scoring System to Predict Survival Outcome of Resectable Colorectal Liver Metastases in this Modern Era. Ann. Surg. Oncol..

[65] Takeda Y., Mise Y., Matsumura M., Hasegawa K., Yoshimoto J., Imamura H., Noro T., Yamamoto J., Ishizuka N., Inoue Y. (2021). Accuracy of modern clinical risk score including RAS status changes based on whether patients received perioperative chemotherapy for colorectal liver metastases. World J. Surg..

[66] Kawaguchi Y., Kopetz S., Tran Cao H.S., Panettieri E., De Bellis M., Nishioka Y., Hwang H., Wang X., Tzeng C.D., Chun Y.S. (2021). Contour prognostic model for predicting survival after resection of colorectal liver metastases: Development and multicentre validation study using largest diameter and number of metastases with RAS mutation status. Br. J. Surg..

[67] Sasaki K., Gagnière J., Dupré A., Ardiles V., O’Connor J.M., Wang J., Moro A., Morioka D., Buettner S., Gau L. (2021). Performance of two prognostic scores that incorporate genetic information to predict long-term outcomes following resection of colorectal cancer liver metastases: An external validation of the MD Anderson and JHH-MSK scores. J. Hepatobiliary Pancreat. Sci..

[68] Chen Y., Chang W., Ren L., Chen J., Tang W., Liu T., Jian M., Liu Y., Wei Y., Xu J. (2020). Comprehensive evaluation of relapse risk (CERR) score for colorectal liver metastases: Development and validation. Oncologist.

[69] Kim W.J., Lim T.W., Kang S.H., Park P.J., Choi S.B., Lee S.I., Min B.W., Kim W.B. (2020). Development and validation of novel scoring system for the prediction of disease recurrence following resection of colorectal liver metastasis. Asian J. Surg..

[70] Liu W., Wang K., Han Y., Liang J.Y., Li Y.H., Xing B.C. (2019). Nomogram predicted disease free survival for colorectal liver metastasis patients with preoperative chemotherapy followed by hepatic resection. Eur. J. Surg. Oncol..

[71] Dupré A., Berhane S., Chan A.W.H., Rivoire M., Chong C.C.N., Lai P.B.S., Cucchetti A., Poston G.J., Malik H.Z., Johnson P.J. (2019). Multicentre validation of a clinical prognostic score integrating the systemic inflammatory response to the host for patients treated with curative-intent for colorectal liver metastases: The Liverpool score. Eur. J. Surg. Oncol..

[72] Bolhuis K., Wensink G.E., Elferink M.A.G., Bond M.J.G., Dijksterhuis W.P.M., Fijneman R.J.A., Kranenburg O.W., Rinkes I.H.M.B., Koopman M., Swijnenburg R.J. (2022). External Validation of Two Established Clinical Risk Scores Predicting Outcome after Local Treatment of Colorectal Liver Metastases in a Nationwide Cohort. Cancers.

[73] Bai L., Lin Z.Y., Lu Y.X., Chen Q., Zhou H., Meng Q., Lin C.P., Huang W.L., Wan Y.L., Pan Z.Z. (2021). The prognostic value of preoperative serum lactate dehydrogenase levels in patients underwent curative-intent hepatectomy for colorectal liver metastases: A two-center cohort study. Cancer Med..

[74] Meng Q., Zheng N., Wen R., Sui J., Zhang W. (2021). Preoperative nomogram to predict survival following colorectal cancer liver metastasis simultaneous resection. J. Gastrointest. Oncol..

[75] Paredes A.Z., Hyer J.M., Tsilimigras D.I., Moro A., Bagante F., Guglielmi A., Ruzzenente A., Alexandrescu S., Makris E.A., Poultsides G.A. (2020). A Novel Machine-Learning Approach to Predict Recurrence After Resection of Colorectal Liver Metastases. Ann. Surg. Oncol..

[76] Gasser E., Braunwarth E., Riedmann M., Cardini B., Fadinger N., Presl J., Klieser E., Ellmerer P., Dupré A., Imai K. (2019). Primary tumour location affects survival after resection of colorectal liver metastases: A two-institutional cohort study with international validation, systematic meta-analysis and a clinical risk score. PLoS ONE.

[77] Dai S., Ye Y., Kong X., Li J., Ding K. (2021). A predictive model for early recurrence of colorectal-cancer liver metastases based on clinical parameters. Gastroenterol. Rep..

[78] Gregoire E., Hoti E., Gorden D.L., de la Serna S., Pascal G., Azoulay D. (2010). Utility or futility of prognostic scoring systems for colorectal liver metastases in an era of advanced multimodal therapy. Eur. J. Surg. Oncol..

[79] Schadde E., Grunhagen D.J., Verhoef C., Krzywon L., Metrakos P. (2021). Limitations in resectability of colorectal liver metastases 2020—A systematic approach for clinicians and patients. Semin. Cancer Biol..

[80] Viganò L., Darwish S.S., Rimassa L., Cimino M., Carnaghi C., Donadon M., Procopio F., Personeni N., Del Fabbro D., Santoro A. (2018). Progression of Colorectal Liver Metastases from the End of Chemotherapy to Resection: A New Contraindication to Surgery?. Ann. Surg. Oncol..

[81] Haddad A., Lendoire M., Uppal A., Maki H., Folkert I., Wang Y., Ayabe R.I., Newhook T.E., Chun Y.S., Tzeng C.D. (2025). CEA Rebound After Discontinuation of Pre-Hepatectomy Chemotherapy Predicts Worse Outcomes After Resection of Colorectal Cancer Liver Metastases. Ann. Surg. Oncol..

[82] Ayez N., van der Stok E.P., de Wilt H., Radema S.A., van Hillegersberg R., Roumen R.M., Vreugdenhil G., Tanis P.J., Punt C.J., Dejong C.H. (2015). Neo-adjuvant chemotherapy followed by surgery versus surgery alone in high-risk patients with resectable colorectal liver metastases: The CHARISMA randomized multicenter clinical trial. BMC Cancer.

[83] Creasy J.M., Sadot E., Koerkamp B.G., Chou J.F., Gonen M., Kemeny N.E., Balachandran V.P., Kingham T.P., DeMatteo R.P., Allen P.J. (2018). Actual 10-year survival after hepatic resection of colorectal liver metastases: What factors preclude cure?. Surgery.

[84] Dueland S., Smedman T.M., Syversveen T., Grut H., Hagness M., Line P.D. (2023). Long-Term Survival, Prognostic Factors, and Selection of Patients With Colorectal Cancer for Liver Transplant: A Nonrandomized Controlled Trial. JAMA Surg..

[85] van de Geest T.W., van Amerongen M.J., Nierop P.M.H., Höppener D.J., Grünhagen D.J., Moelker A., Fütterer J.J., Verhoef C., de Wilt J.H.W. (2022). Propensity score matching demonstrates similar results for radiofrequency ablation compared to surgical resection in colorectal liver metastases. Eur. J. Surg. Oncol..

[86] Nakai T., Ishikawa H., Tokoro T., Okuno K. (2015). The clinical risk score predicts the effectiveness of adjuvant chemotherapy for colorectal liver metastasis. World J. Surg..

[87] Giuliante F., Viganò L., De Rose A.M., Mirza D.F., Lapointe R., Kaiser G., Barroso E., Ferrero A., Isoniemi H., Lopez-Ben S. (2021). Liver-First Approach for Synchronous Colorectal Metastases: Analysis of 7360 Patients from the LiverMetSurvey Registry. Ann. Surg. Oncol..

[88] Viganò L., Capussotti L., De Rosa G., De Saussure W.O., Mentha G., Rubbia-Brandt L. (2013). Liver resection for colorectal metastases after chemotherapy: Impact of chemotherapy-related liver injuries, pathological tumor response, and micrometastases on long-term survival. Ann. Surg..

[89] Görgec B., Verpalen I.M., Sijberden J.P., Abu Hilal M., Bipat S., Verhoef C., Swijnenburg R.J., Besselink M.G., Stoker J. (2024). Added Value of Liver MRI in Patients Eligible for Surgical Resection or Ablation of Colorectal Liver Metastases Based on CT: A Systematic Review and Meta-Analysis. Ann. Surg. Open.

[90] Mlecnik B., Van den Eynde M., Bindea G., Church S.E., Vasaturo A., Fredriksen T., Lafontaine L., Haicheur N., Marliot F., Debetancourt D. (2018). Comprehensive intrametastatic immune quantification and major impact of immunoscore on survival. J. Natl. Cancer Inst..

[91] Lambin P., Leijenaar R.T.H., Deist T.M., Peerlings J., de Jong E.E.C., van Timmeren J., Sanduleanu S., Larue R.T.H.M., Even A.J.G., Jochems A. (2017). Radiomics: The bridge between medical imaging and personalized medicine. Nat. Rev. Clin. Oncol..

[92] Viganò L., Ammirabile A., Zwanenburg A. (2023). Radiomics in liver surgery: Defining the path toward clinical application. Updates Surg..

[93] Fiz F., Viganò L., Gennaro N., Costa G., La Bella L., Boichuk A., Cavinato L., Sollini M., Politi L.S., Chiti A. (2020). Radiomics of Liver Metastases: A Systematic Review. Cancers.

[94] Giuliante F., Ardito F., Vellone M., Ranucci G., Federico B., Giovannini I., Nuzzo G. (2009). Role of the surgeon as a variable in long-term survival after liver resection for colorectal metastases. J. Surg. Oncol..

[95] Dorcaratto D., Mazzinari G., Fernandez M., Muñoz E., Garcés-Albir M., Ortega J., Sabater L. (2019). Impact of Postoperative Complications on Survival and Recurrence After Resection of Colorectal Liver Metastases: Systematic Review and Meta-analysis. Ann. Surg..

[96] Viganò L., Procopio F., Cimino M.M., Donadon M., Gatti A., Costa G., Del Fabbro D., Torzilli G. (2016). Is Tumor Detachment from Vascular Structures Equivalent to R0 Resection in Surgery for Colorectal Liver Metastases? An Observational Cohort. Ann. Surg. Oncol..

[97] Rubbia-Brandt L., Giostra E., Brezault C., Roth A.D., Andres A., Audard V., Sartoretti P., Dousset B., Majno P.E., Soubrane O. (2007). Importance of histological tumor response assessment in predicting the outcome in patients with colorectal liver metastases treated with neo-adjuvant chemotherapy followed by liver surgery. Ann. Oncol..

[98] Viganò L., Branciforte B., Laurenti V., Costa G., Procopio F., Cimino M., Del Fabbro D., Di Tommaso L., Torzilli G. (2022). The histopathological growth pattern of colorectal liver metastases impacts local recurrence risk and the adequate width of the surgical margin. Ann. Surg. Oncol..

[99] Yamashita S., Chun Y.S., Kopetz S.E., Vauthey J.N. (2018). Biomarkers in colorectal liver metastases. Br. J. Surg..

[100] Lang H., Baumgart J., Heinrich S., Tripke V., Passalaqua M., Maderer A., Galle P.R., Roth W., Kloth M., Moehler M. (2019). Extended molecular profiling improves stratification and prediction of survival after resection of colorectal liver metastases. Ann. Surg..

[101] Yamashita S., Chun Y.S., Kopetz S.E., Maru D., Conrad C., Aloia T.A., Vauthey J.N. (2020). APC and PIK3CA mutational cooperativity predicts pathologic response and survival in patients undergoing resection for colorectal liver metastases. Ann. Surg..

