# Supplementary material for: Prognostic Scores for Liver Resection in Colorectal Metastases: Performance, Limitations, and Methodological Pitfalls—A Systematic Review and Meta-Analysis
Source: Cancers (Basel). 2026 Feb 14;18(4):625. doi: 10.3390/cancers18040625 (PMC12939581; doi:10.3390/cancers18040625)

Supplementary Figure S1. Funnel plots for each prognostic score included in the meta-analysis.

a. Fong OS C-index

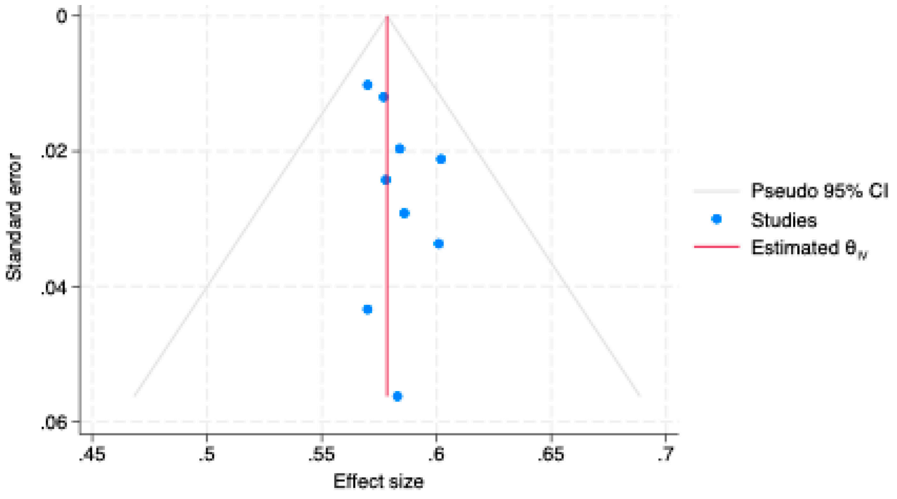

b. Fong OS 1-year C-index

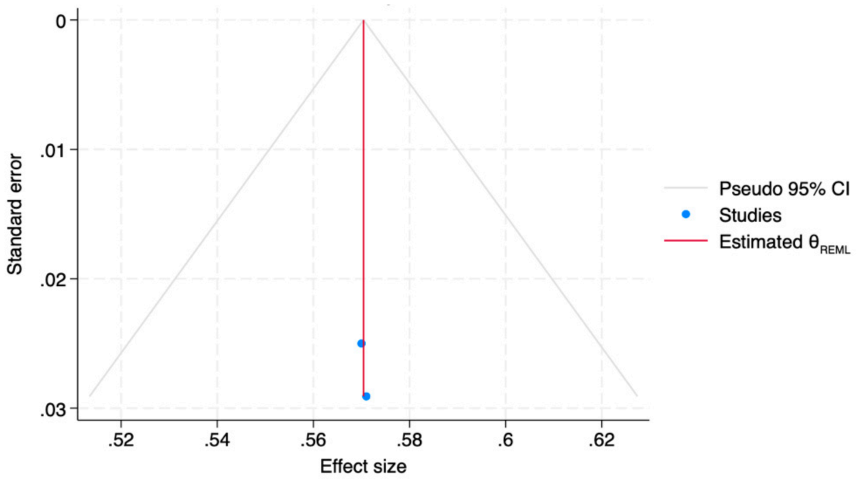

c. Fong OS 3-year C-index

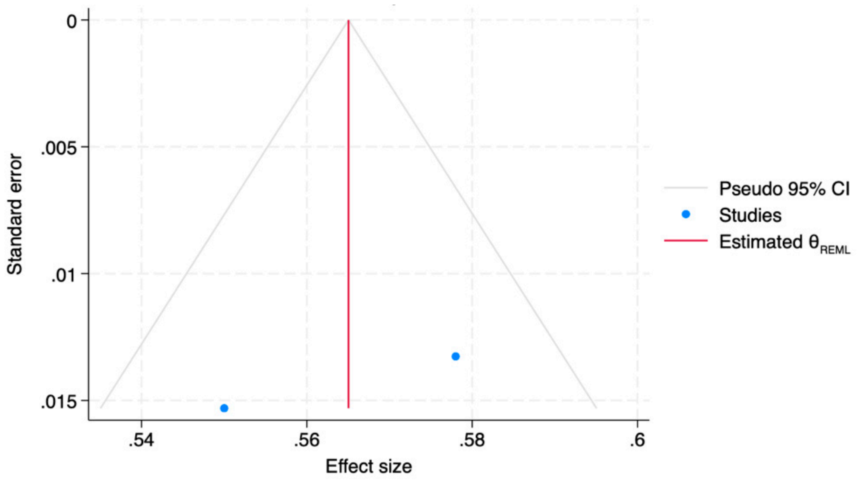

d. Fong OS 5-year C-index

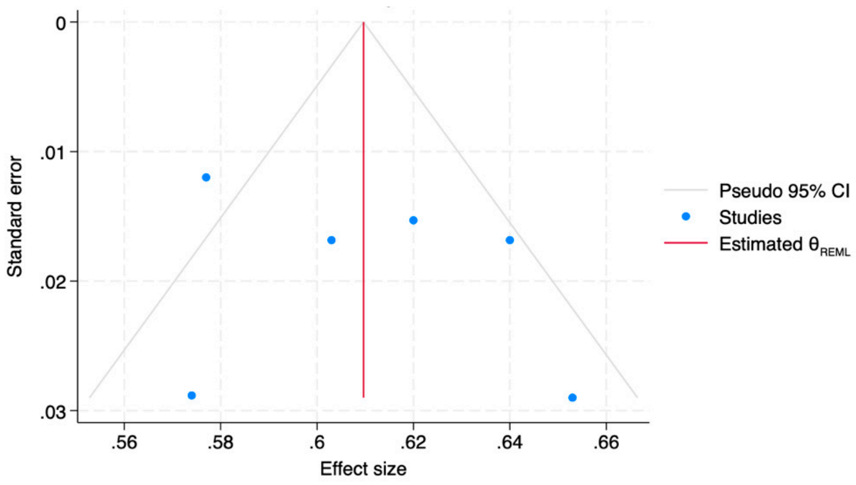

e. Fong OS AUC

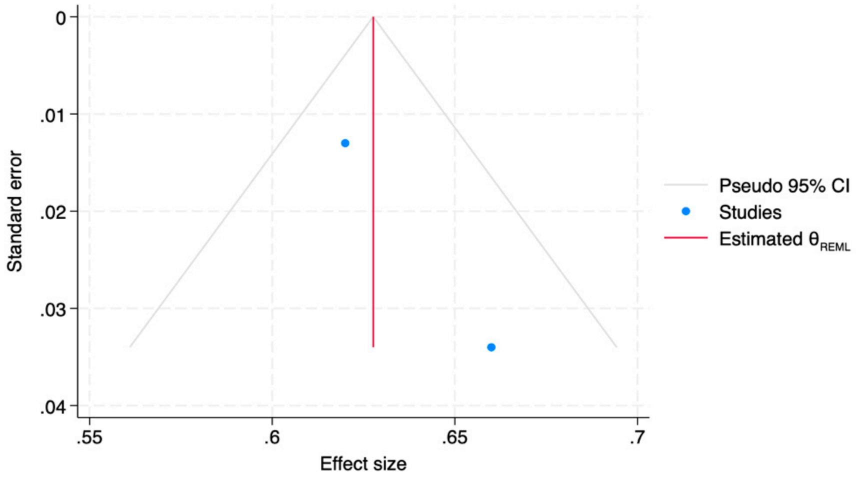

f. Fong OS 1-year AUC

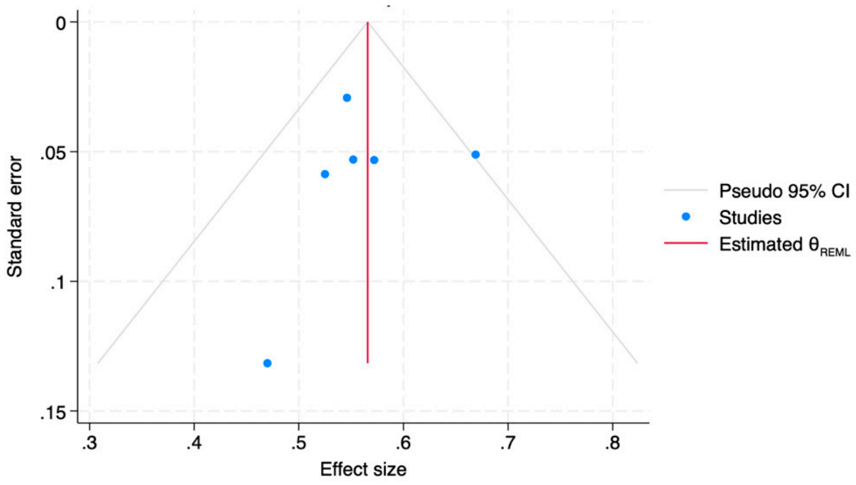

g. Fong OS 3-year AUC

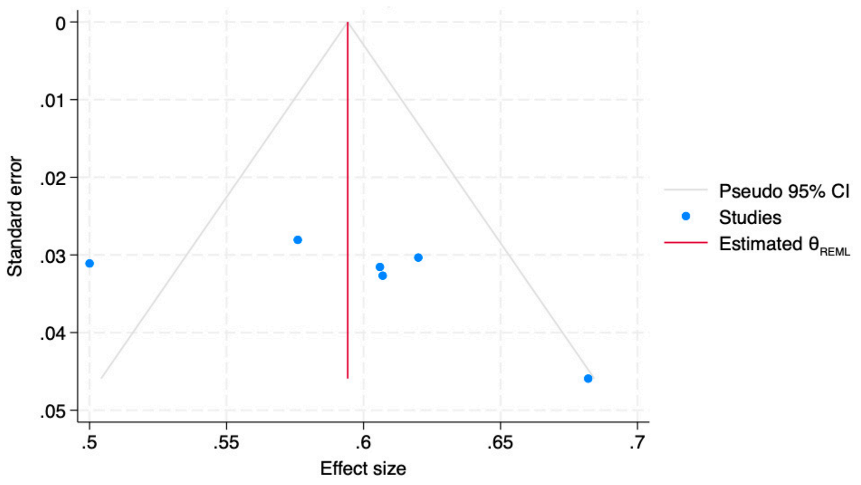

h. Fong OS 5-year AUC

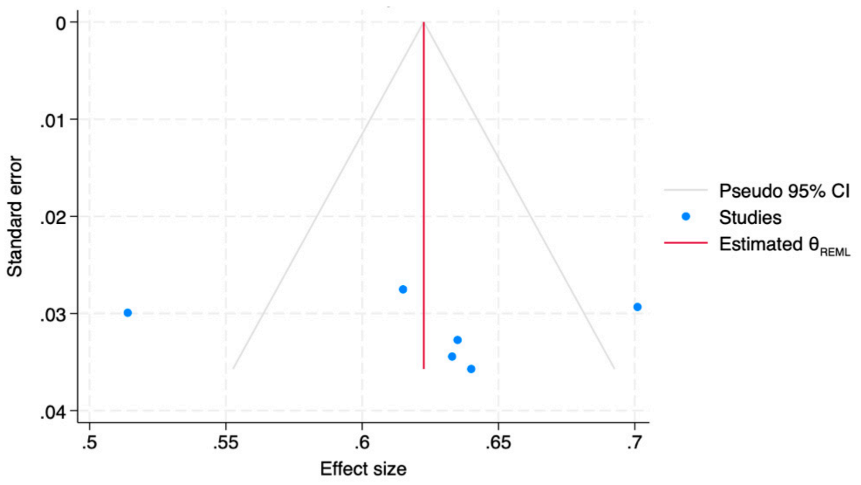

i. Fong DFS C-index

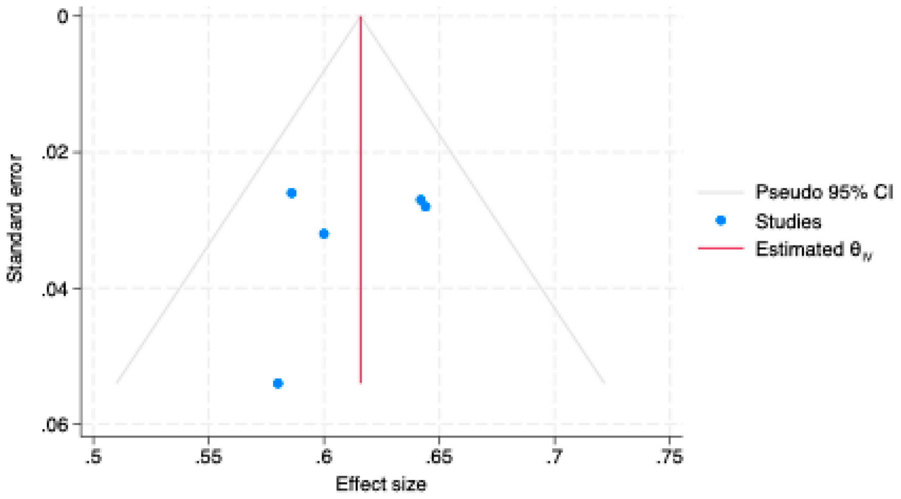

j. Fong DFS AUC

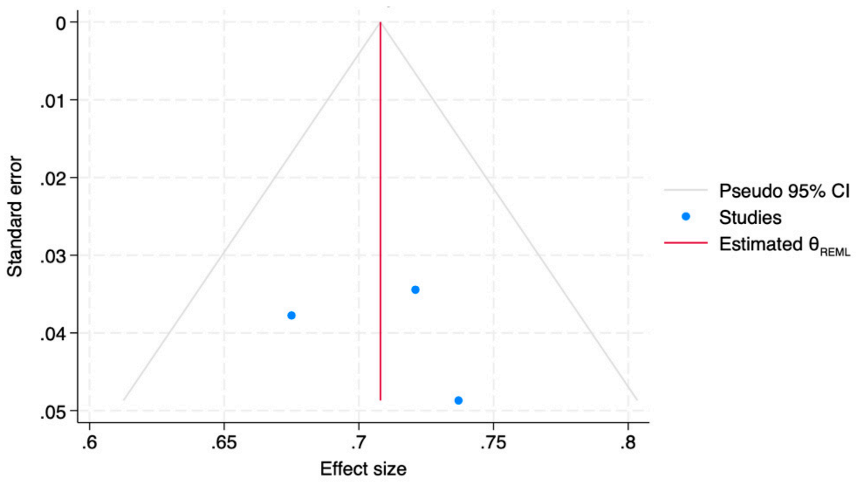

k. Fong DFS 1-year AUC

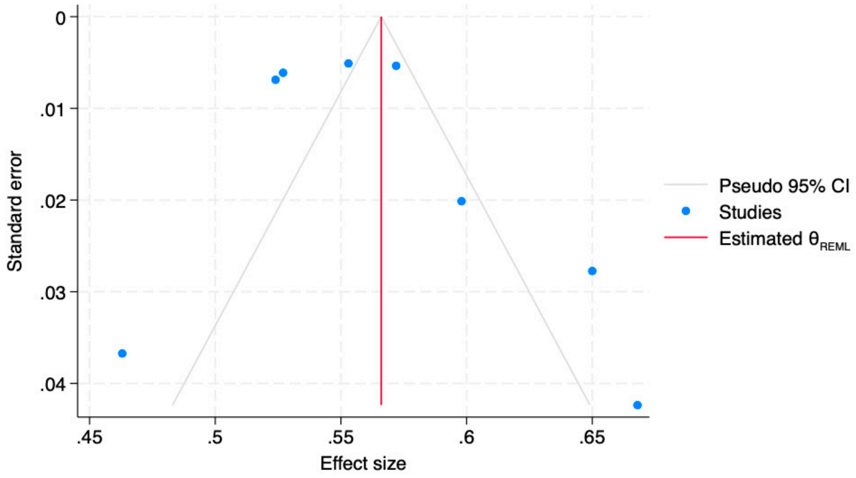

l. Fong DFS 3-year AUC

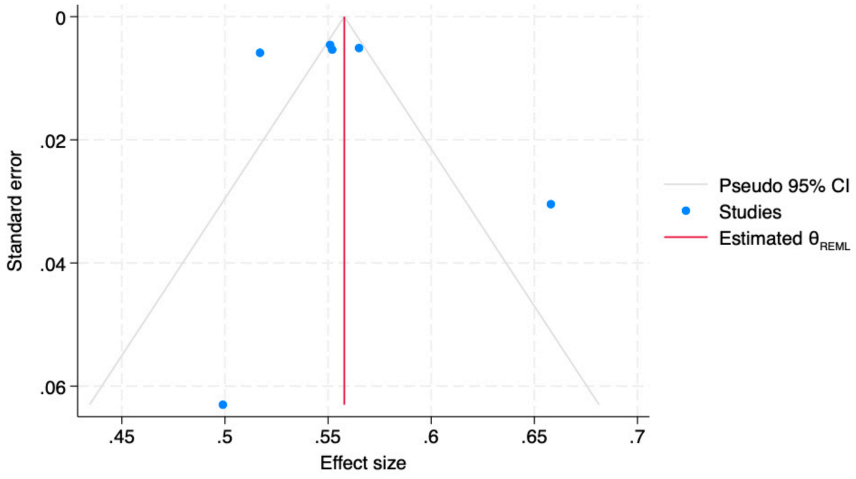

m. Fong DFS 5-year AUC

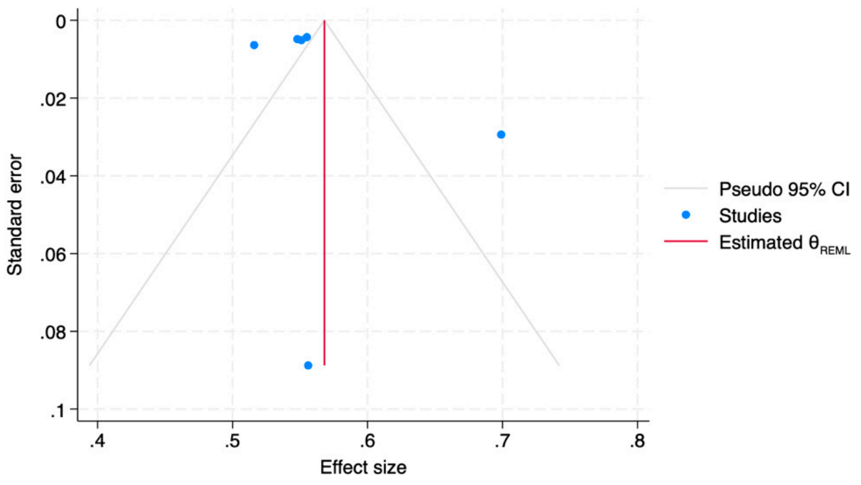

n. GAME OS C-index

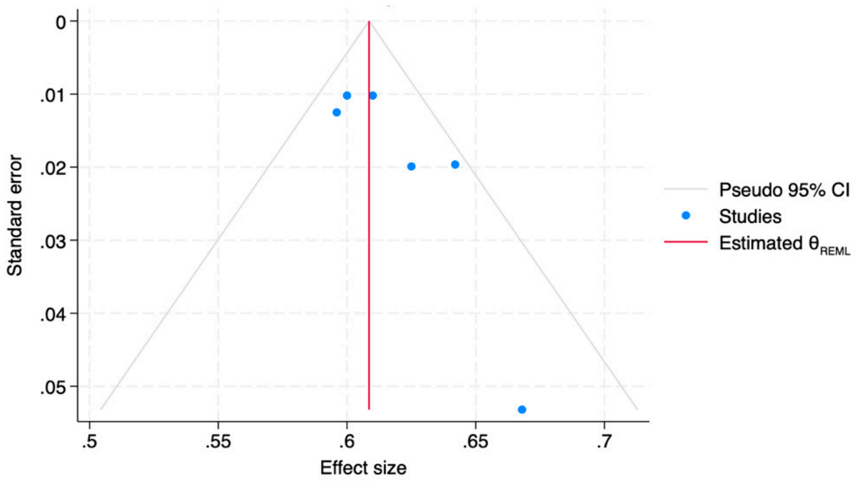

o. RASmut-CRS OS C-index

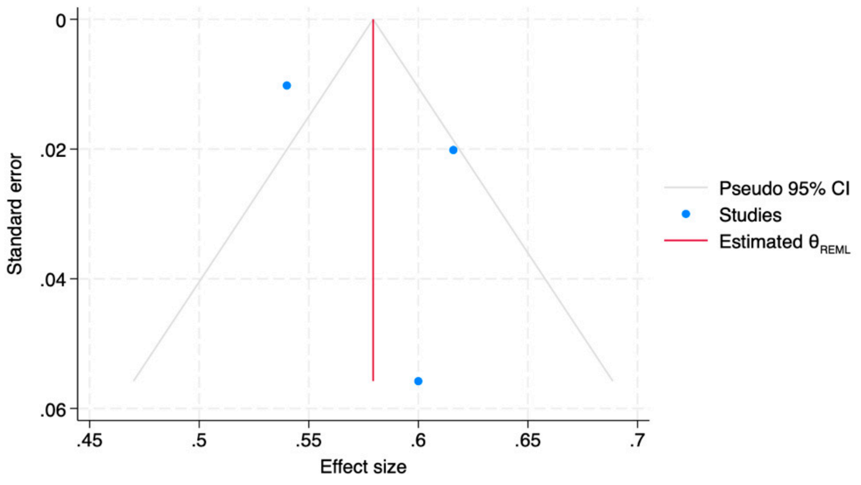

p. RASmut-CRS OS 1-year AUC

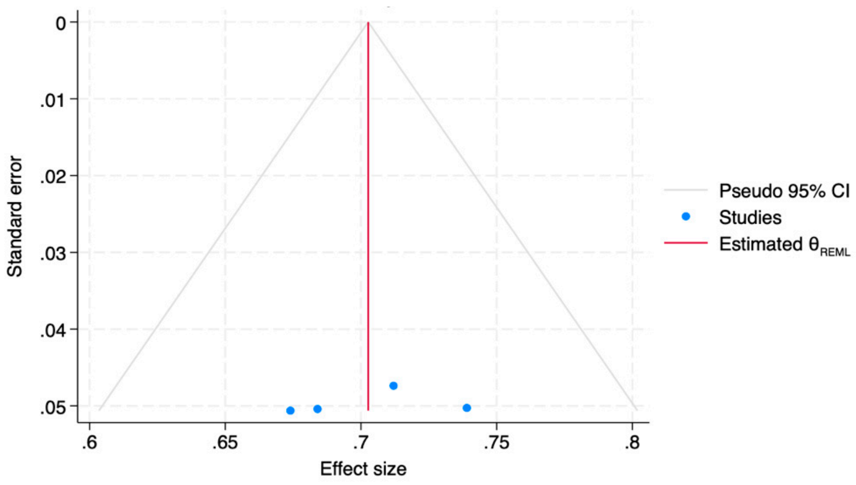

q. RASmut-CRS OS 3-years AUC

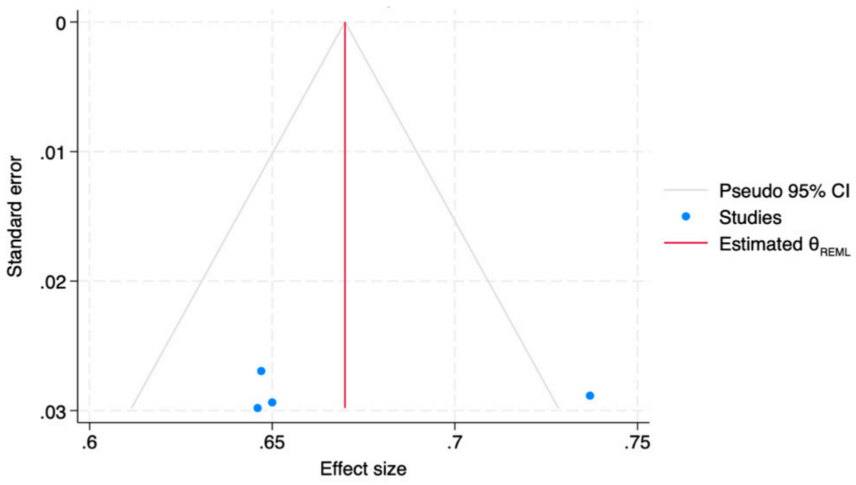

r. RASmut-CRS OS 5-years AUC

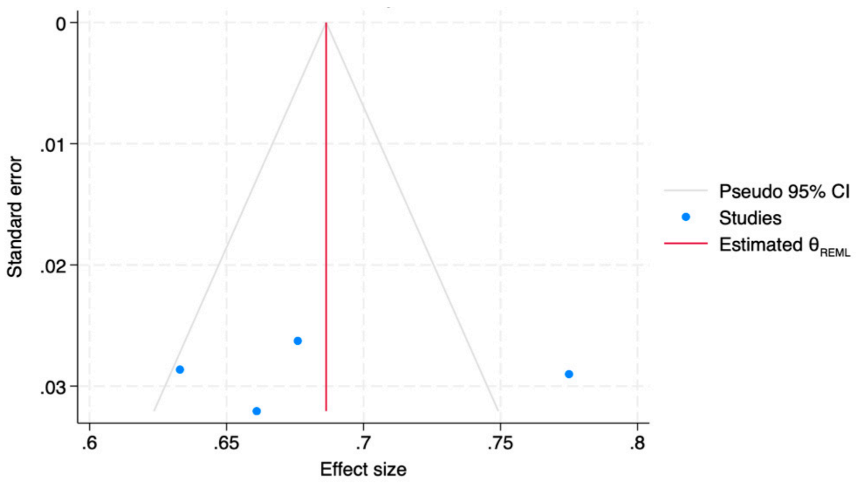

Supplement: Supplementary file 1 [file cancers-18-00625-s001.zip › Supplementary Figure 1.pdf]
