# Supplementary material for: Prognostic Scores for Liver Resection in Colorectal Metastases: Performance, Limitations, and Methodological Pitfalls—A Systematic Review and Meta-Analysis
Source: Cancers (Basel). 2026 Feb 14;18(4):625. doi: 10.3390/cancers18040625 (PMC12939581; doi:10.3390/cancers18040625)
Supplement: Supplementary file 1 [file cancers-18-00625-s001.zip › Supplementary Table 2.pdf]

Supplementary Table S2. Patient characteristics. *If multiple cohorts of patients are analyzed in a single paper, multiple lines are reported (one for each cohort).*

Author	#	Age		Female patients n (%)	Synchronous metastases n (%)	Primary rectal cancer n (%)	Size (mm)			Number of metastases		Preop. Chemo n (%)
		mean (SD)	≥ 65 y				mean (SD)	> 50 mm n (%)	≥ 50 mm n (%)	Solitary n (%)	mean (SD)	
Skipenko OG et al.	312	57,9 (16)		154 (49)	176 (56)	131 (42)		106 (34)		112 (36)		87 (28)
Sasaki K et al.	430						31,7 (18,5)				3 (3,7)	193 (45)
	198						33,3 (22,2)				3 (3,7)	91 (46)
Wang Y et al.	249		46 (18)	91 (37)	55 (22)	90 (36)		52 (21)		90 (36)		144 (58)
Ding Y et al.	102		74 (73)[b]	24 (24)	102 (100)	53 (52)			37 (36)			102 (100)
Chen Q et al.	230	56,6 (10,6)		98 (43)	230 (100)	32 (14)	27 (17)				4,5 (4,5)	196 (85)
Jiang C et al.	371		160 (43)[c]	129 (35)	171 (46)			66 (18)		233 (63)		175 (47)
Martin-Cullell B et al.	176	68,3 (10,4)		59 (34)	100 (57)	36 (21)	25,3 (17)				2 (1,4)	50 (35)
Takematsu T et al.	218		39 (18)[g]	79 (36)	143 (66)			26 (12)		74 (34)		117 (54)
Qi L et al.	433	56,7 (11,9)		141 (33)	290 (67)		28,7 (16,3)				2,3 (2,2)	274 (63)
	404	56,7 (11,9)		129 (32)	211 (52)		28,3 (14,8)				2,3 (2,2)	293 (73)
Lam CSN et al.	172	63,3 (9,6)		59 (34)	100 (58)	65 (38)	28,7 (19,3)				1,6 (1,4)	30 (18)
Katipally RR et al.	147	64 (7,4)		50 (34)	106 (72)			38 (26)		35 (24)		147 (100)
Li T et al.	122		117 (96) **	42 (34)	122 (100)	43 (35)		8 (7)				101 (83)
Reijonen P et al.	816	61 (10,2)		321 (39)	486 (60)	307 (38)			106 (13)		1,6 (1,4)	598 (73)
Chen J et al.	28	62 (9)		8 (29)					7 (25)	14 (50)		28 (100)
Zhang C et al.	106	57,3 (14,3)		40 (38)	64 (60)				32 (30)	44 (42)		58 (55)
	95	58,3 (13,3)		37 (39)	55 (58)				25 (26)	61 (64)		44 (46)
Beppu T et al.	1756				800 (46)			253 (14)		995 (57)		0
	469				315 (67)			105 (23)		142 (30)		469 (100)
Chen Q et al.	389	57 (10,4)		144 (38)	290 (77)	175 (46)		45 (12)		176 (46)		196 (52)
Bao X et al.	312											
	144		32 (22)	52 (36)	91 (63)			12 (8)		50 (35)		116 (81)
Chen FL et al.	375		180 (48)[d]	132 (35)	241 (64)				66 (18)	375 (100)		193 (51)
	424		206 (49)[d]	160 (38)	365 (86)				49 (12)	0		352 (83)
	296		103 (35)[d]	94 (32)	285 (96)				22 (7)	0		282 (95)
Villard C et al.	391	58,8 (9,8)		145 (37)	274 (70)			108 (28)		166 (43)^		321 (82)
	1013	62,3 (11,2)		381 (38)	722 (71)			185 (18)		441 (44)^		716 (71)
Zhou Z et al.	118		39 (33)[c]	39 (33)	92 (78)			29 (25)		43 (36)		118 (100)
Filippini Velazquez G et al.	230	61,3 (10,2)		67 (29)	133 (58)						2,7 (0,5)	81 (35)
Buisman FE et al.	3064	64,3 (9,6)		367 (35)	734 (40)	379 (37)		163 (17)		452 (44)		486 (47)
	1048	59,3 (13,3)		1323 (43)	2085 (68)	725 (25)		729 (24)		1252 (41)		2556 (91)
	4112	60,7 (12,6)		1690 (41)	2819 (69)	1104 (28)		892 (22)		1704 (42)		3042 (79)
Paro A et al.	672	60,3 (11,9)		406 (60)	442 (66)	189 (28)	32,7 (20,7)				2 (1,4)	367 (55)
Bai L et al.	325	54,5 (9,3)		111 (34)	174 (54)			50 (15)		140 (43)		207 (64)
	341	55,5 (9,3)		113 (33)	233 (68)			59 (17)		167 (47)		179 (52)
Sasaki K et al.	1307	62 (11,9)		470 (36)	1096 (84)	259 (20)	25,7 (17)				2,3 (2,2)	494 (38)
	1058	61,7 (12,6)		546 (51)	785 (77)	104 (10)	35 (22,2)				1,3 (0,7)	805 (78)
	2365			1016 (43)	1881 (80)	363 (15)						1299 (55)
	1205	58,7 (12,6)		487 (40)	843 (79)	170 (14)	30,7 (20)				2 (1,4)	873 (72)
Furukawa K et al.	149	62,5 (10,3)		46 (31)	92 (62)		29 (19,3)			71 (48)		51 (34)
Wong GYM et al.	103	64,7 (11,1)		40 (38)	55 (53)	32 (31)	33,3 (22,2)				2 (1,4)	88 (85)
Zhai Y et al.	147		67 (46)[c]	57 (39)	98 (67)	78 (53)	30 (14,8)				3,3 (2,2)	0
Wada Y et al.	151	61,8 (11,5)		50 (33)	98 (65)	56 (37)	54,8 (26,2)			62 (41)		83 (55)
Fruhling P et al.	1212	66,5 (10,4)		464 (38)	700 (58)	498 (41)	28,3 (14,8)			477 (39)^		
Guo X et al.	112		94 (84)[a]	44 (39)	112 (100)	32 (29)		45 (40)				86 (77)
Liu W et al.	237	56,1 (11,4)		87 (37)	220 (93)	74 (31)	31,9 (19,6)				4,7 (1,1)	145 (61)

	532	57,2 (10,1)		191 (36)	440 (83)	203 (38)	30,9 (20,4)				4,2 (1)	324 (61)
Takeda Y et al.	309				245 (79)				57 (18)	85 (28)		71 (33)
Kawaguchi Y et al.	254	63,7 (11,1)		247 (37)	491 (73)	212 (32)	27,7 (18,5)				2,3 (2,2)	422 (63)
	419	63,7 (11,1)		247 (37)	491 (73)	212 (32)	27,7 (18,5)				2,3 (2,2)	422 (63)
Sasaki K et al.	2376	59 (20)		939 (40)	1647 (69)	646 (27)	31,7 (18,5)				2 (1,4)	1821 (77)
Chen Y et al.	787	59,4 (10,5)		246 (31)	621 (79)	313 (40)			204 (26)	306 (39)		
	162				134 (83)				37 (23)	48 (30)		
Kim WJ et al.	295		70 (24)[f]	96 (33)	231 (78)	118 (40)			50 (17)	263 (89)*		56 (19)
Liu W et al.	117	54,4 (11,1)		38 (32)	107 (91)	26 (22)	33,1 (22,5)				3 (8,7)	117 (100)
Duprè A et al.	219									117 (53)		
Margonis GA et al.	747	58 (13,3)			583 (78)		27,7 (19,3)				2,3 (2,2)	
	502	58 (13,3)		206 (41)	370 (74)	106 (21)	27,7 (18,5)				2,3 (2,2)	335 (67)
Brudvik KW et al.	608				358 (59)			120 (20)		234 (39)		
	564	56 (11)		237 (42)	390 (69)	125 (22)		63 (11)		234 (39)		492 (87)
Bolhuis K et al.	1105	65,7 (9,6)		415 (38)	823 (75)	371 (34)	25,7 (14,8)				2,3 (2,2)	393 (36)
Bai L et al.	580	55 (10,3)		195 (34)	440 (76)			80 (14)		262 (45)		252 (43)
Meng Q et al.	174		52 (30)	74 (43)	174 (100)				29 (17)	90 (52)		47 (27)
	60		18 (30)	34 (57)	60 (100)				10 (17)	29 (48)		21 (35)
Paredes AZ et al.	703	60,3 (11,9)		286 (41)		211 (30)	33,3 (22,2)				2 (1,4)	387 (55)
	703	60,7 (11,1)		271 (39)		205 (29)	33 (23)				2 (1,4)	388 (55)
Gasser E et al.	527		373 (71)[c]									
Dai S et al.	202	60,6		62 (31)	143 (71)	66 (33)				149 (74)°		

a > 50; b > 57; c > 60; d ≥ 60; e > 65; f > 70; g ≥ 75; ** >= 40 yrs; ^ <3; ° ≤4: *<5 metastases
