# Supplementary material for: Prognostic Scores for Liver Resection in Colorectal Metastases: Performance, Limitations, and Methodological Pitfalls—A Systematic Review and Meta-Analysis
Source: Cancers (Basel). 2026 Feb 14;18(4):625. doi: 10.3390/cancers18040625 (PMC12939581; doi:10.3390/cancers18040625)
Supplement: Supplementary file 1 [file cancers-18-00625-s001.zip › Supplementary Table 3.pdf]

Supplementary Table S3. Quality assessment according to the Prediction Model Risk of Bias Assessment Tool (PROBAST)

Author	Participants		Predictors		Outcome		Analysis	Overall	
	**A)**	**B)**	**A)**	**B)**	**A)**	**B)**	**A)**	**A)**	**B)**
Skipenko OG et al.	Low	Low	Low	Low	Low	Low	Low	Low	Low
Sasaki K et al.	Low	Low	Low	Low	Low	Low	High	High	Low
Wang Y et al.	Low	Low	Low	Low	Low	Low	Low	Low	Low
Ding Y et al.	Low	High	Low	Low	Low	Low	Low	Low	High
Chen Q et al.	Low	High	Low	Low	Low	Low	High	High	High
Jiang C et al.	Low	Low	Low	Low	Low	Low	High	High	Low
Martin-Cullell B et al.	Low	Low	Low	Low	Low	Low	High	High	Low
Takematsu T et al.	Low	Low	Low	Low	Low	Low	High	High	Low
Qi L et al.	Low	Low	Low	Low	Low	Low	Low	Low	Low
Lam CSN et al.	Low	Low	Low	Low	Low	Low	Low	Low	Low
Katipally RR et al.	Low	Low	Low	Low	Low	Low	Low	Low	Low
Li T et al.	Low	High	Low	Low	Low	Low	High	High	High
Reijonen P et al.	Low	Low	Low	Low	Low	Low	High	High	Low
Chen J et al.	High	High	Low	Low	Low	Low	High	High	High
Zhang C et al.	Low	Low	Low	Low	Low	Low	Low	Low	Low
Beppu T et al.	Low	Low	Low	Low	Low	Low	Low	Low	Low
Chen Q et al.	Low	Low	Low	Low	Low	Low	High	High	Low
Bao X et al.	High	High	Low	Low	Low	Low	High	High	High
Chen FL et al.	High	Low	Low	Low	Low	Low	High	High	Low
Villard C et al.	High	High	Low	Low	Low	Low	High	High	High
Zhou Z et al.	Low	Low	Low	Low	Low	Low	High	High	Low
Filippini Velazquez G et al.	Low	Low	Low	Low	Low	Low	High	High	Low
Buisman FE et al.	Low	Low	Low	Low	Low	Low	Low	Low	Low
Paro A et al.	Low	Low	Low	Low	Low	Low	High	High	Low

Bai L et al.	Low	Low	Low	Low	Low	Low	Low	Low	Low
Sasaki K et al.	Low	Low	Low	Low	Low	Low	High	High	Low
Furukawa K et al.	Low	Low	Low	High	Low	Low	High	High	High
Wong GYM et al.	Low	Low	Low	Low	Low	Low	High	High	Low
Zhai Y et al.	Low	Low	Low	Low	Low	Low	High	High	Low
Wada Y et al.	Low	Low	Low	High	Low	Low	Low	Low	High
Fruhling P et al.	Low	Low	Low	Low	Low	Low	High	High	Low
Guo X et al.	Low	High	Low	Low	Low	Low	Low	Low	High
Liu W et al.	Low	Low	Low	Low	Low	Low	High	High	Low
Takeda Y et al.	Low	Low	Low	Low	Low	Low	High	High	Low
Kawaguchi Y et al.	Low	Low	Low	Low	Low	Low	Low	Low	Low
Sasaki K et al.	Low	Low	Low	Low	Low	Low	Low	Low	Low
Chen Y et al.	Low	Low	Low	Low	Low	Low	Low	Low	Low
Kim WJ et al.	Low	Low	Low	Low	Low	Low	Low	Low	Low
Liu W et al.	Low	Low	Low	Low	Low	Low	High	High	Low
Duprè A et al.	Low	High	Low	Low	Low	Low	Low	Low	High
Margonis GA et al.	Low	Low	Low	Low	Low	Low	Low	Low	Low
Brudvik KW et al.	Low	Low	Low	Low	Low	Low	High	High	Low
Bolhuis K et al.	Low	High	Low	Low	Low	Low	Low	Low	High
Bai L et al.	Low	Low	Low	Low	High	Low	Low	High	Low
Meng Q et al.	High	High	Low	Low	Low	Low	High	High	High
Paredes AZ et al.	Low	Low	Low	Low	Low	Low	Low	Low	Low
Gasser E et al.	Low	Low	High	Low	Low	Low	High	High	Low
Dai S et al.	High	Low	Low	Low	Low	High	Low	High	High

A: Risk of bias
B: Concern of applicability
