# Supplementary material for: Prognostic Scores for Liver Resection in Colorectal Metastases: Performance, Limitations, and Methodological Pitfalls—A Systematic Review and Meta-Analysis
Source: Cancers (Basel). 2026 Feb 14;18(4):625. doi: 10.3390/cancers18040625 (PMC12939581; doi:10.3390/cancers18040625)
Supplement: Supplementary file 1 [file cancers-18-00625-s001.zip › Supplementary Table 4.pdf]

Supplementary Table S4. Performances of the score for OS prediction.

Analyzed score	Author	# patients	Metric type	Value	95%CI
Author's immunoscore	Zhang C et al.	106	C-index	0,750	0,639-0,862
		95	C-index	0,732	0,610-0,854
Author's nomogram	Guo X et al.	112	C-index	0,670	0,588-0,752
		112	1-year AUC	0,814	-
		112	3-years AUC	0,694	-
		112	5-years AUC	0,611	-
Author's nomogram	Ding Y et al.	102	C-index	0,671	0,591-0,751
		102	1-year C-index	0,623	-
		102	3-years C-index	0,682	-
		102	5-years C-index	0,703	-
Author's nomogram	Li T et al.	122	1-year AUC	0,755	-
		122	2-years AUC	0,885	-
		122	3-years AUC	0,908	-
Author's radiomic and clinical score	Chen J et al.	28	C-index	0,740	-
Author’s radiomic score	Chen J et al.	28	C-index	0,640	-
		28	C-index	0,630	-
Author's score	Filippini Velazquez G et al.	230	C-index	0,676	-
Author's score	Qi L et al.	404	p-value	< 0,001	-
Author's score	Sasaki K et al.	1307	C-index	0,620	-
		1307	C-index	0,600	-
		1058	C-index	0,570	-
		1058	C-index	0,570	-
		2365	C-index	0,590	-
		2365	C-index	0,580	-
Author's score	Villard C et al.	391	C-index	0,660	-
Author's score	Bai L et al.	325	5-years C-index	0,665	0,634-0,696
Author's score	Chen Q et al.	230	1-year AUC	0,697	-
		230	3-years AUC	0,692	-
		230	5-years AUC	0,701	-
Author's score	Buisman FE et al.	3064	AUC	0,730	0,68-0,78
		1048	AUC	0,730	0,70-0,75
Author's score	Gasser E et al.	527	p-value	0,001	-
Author's score	Bao X et al.	312	p-value	0,030	-
Author's score	Takeda Y et al.	309	p-value	0,288	-
Beppu score from nomogram	Takematsu T et al.	218	p-value	0,008	-
Contour plot	Kawaguchi Y et al.	254	C-index	0,644	-
		419	C-index	0,624	-
Fong	Bai L et al.	580	5-years C-index	0,653	-
	Bai L et al.	325	5-years C-index	0,603	0,570-0,636
		341	5-years C-index	0,640	0,607-0,673
	Bao X et al.	144	1-year AUC	0,470	-
		144	3-years AUC	0,682	-
		144	5-years AUC	0,628	-
	Bolhuis K et al.	1105	C-index	0,577	0,554-0,601

		1105	1-year C-index	0,570	0,521-0,619
		1105	3-years C-index	0,578	0,552-0,604
		1105	5-years C-index	0,577	0,554-0,601
	Brudvik KW et al.	564	C-index	0,570	0,48-0,65
		608	p-value	< 0,05	-
	Buisman FE et al.	4112	AUC	0,620	0,59-0,64
	Chen FL et al.	375	1-year AUC	0,525	-
		375	3-years AUC	0,500	-
		375	5-years AUC	0,514	-
		424	1-year AUC	0,572	-
		424	3-years AUC	0,576	-
		424	5-years AUC	0,615	-
		296	1-year AUC	0,552	-
		296	3-years AUC	0,607	-
		296	5-years AUC	0,633	-
	Chen Q et al.	389	C-index	0,586	-
		389	1-year AUC	0,546	-
		389	3-years AUC	0,606	-
		389	5-years AUC	0,701	-
	Duprè A et al.	219	C-index	0,601	0,535-0,667
	Jiang C et al.	371	C-index	0,602	0.560-0.643
		371	1-year AUC	0,669	-
		371	3-years AUC	0,620	-
		371	5-years AUC	0,635	-
	Katipally RR et al.	147	K-index	0,590	0,52-0,66
	Lam CSN et al.	172	1-year C-index	0,571	0,514-0,628
		172	5-years C-index	0,574	0,517-0,630
	Margonis GA et al.	747	C-index	0,584	0,545-0,622
		502	C-index	0,578	0,530-0,625
	Meng Q et al.	174	1-year AUC	0,652	-
		174	3-years AUC	0,712	-
		60	1-year AUC	0,591	-
		60	3-years AUC	0,801	-
	Paro A et al.	672	Cohen's D	-	-
	Qi L et al.	433	5-years AUC	0,640	0,57-0,71
		433	8-years AUC	0,670	0,56-0,78
	Reijonen P et al.	816	AUC	0,580	-
	Sasaki K et al.	2365	C-index	0,540	-
		2365	C-index	0,530	-
		1205	C-index	0,570	-
		1205	C-index	0,550	-
	Sasaki K et al.	2376	C-index	0,570	0,55-0,59
	Skipenko OG et al.	312	3-years C-index	0,550	0,52-0,58
		312	5-years C-index	0,620	0,59-0,65
	Takeda Y et al.	309	p-value	0,003	-
	Villard C et al.	1013	C-index	0,590	-
	Wang Y et al.	249	AUC	0,660	0,592-0,727

	Wong GYM et al.	103	C-index	0,583	-
	Zhang C et al.	106	C-index	0,610	-
		95	C-index	0,605	-
Fong with author's score	Qi L et al.	404	p-value	< 0,001	-
Fong with author's score	Sasaki K et al.	2365	C-index	0,590	-
Fong with molecular subtype	Katipally RR et al.	147	K-index	0,630	0,56-0,70
GAME	Bolhuis K et al.	1105	C-index	0,596	0,572-0,621
		1105	1-year C-index	0,583	0,531-0,636
		1105	3-years C-index	0,600	0,573-0,627
		1105	5-years C-index	0,597	0,573-0,621
	Buisman FE et al.	4112	AUC	0,660	0,64-0,69
	Jiang C et al.	371	C-index	0,642	0,604-0,681
		371	1-year AUC	0,713	-
		371	3-years AUC	0,680	-
		371	5-years AUC	0,691	-
	Margonis GA et al.	747	C-index	0,625	0,584-0,662
	Martin-Cullell B et al.	176	C-index	0,620	-
	Paro A et al.	672	Cohen's D	-	-
	Sasaki K et al.	2365	C-index	0,600	-
		2365	C-index	0,580	-
		1205	C-index	0,610	-
		1205	C-index	0,590	-
	Sasaki K et al.	2376	C-index	0,610	0,59-0,63
		2376	C-index	0,600	0,58-0,62
	Villard C et al.	1013	C-index	0,630	-
	Wong GYM et al.	103	C-index	0,668	-
GAME with author's score	Sasaki K et al.	2365	C-index	0,630	-
Integrated clinical-molecular group risk	Katipally RR et al.	147	K-index	0,690	0,63-0,75
	Skipenko OG et al.	312	3-years C-index	0,560	0,50-0,62
		312	5-years C-index	0,680	0,58-0,78
Konopke	Chen FL et al.	375	1-year AUC	0,552	-
		375	3-years AUC	0,529	-
		375	5-years AUC	0,514	-
		424	1-year AUC	0,483	-
		424	3-years AUC	0,525	-
		424	5-years AUC	0,569	-
		296	1-year AUC	0,531	-
		296	3-years AUC	0,494	-
		296	5-years AUC	0,451	-
	Duprè A et al.	219	C-index	0,602	0,543-0,662
Lang-score (ext-ClinScore)	Bao X et al.	144	3-years AUC	0,567	-
		144	5-years AUC	0,586	-
Liverpool score postop	Duprè A et al.	219	C-index	0,637	0,570-0,703
Liverpool score preop	Duprè A et al.	219	C-index	0,619	0,552-0,686
Malik score	Filippini Velazquez G et al.	230	C-index	0,616	-
Modified Glasgow Prognostic score	Furukawa K et al.	149	AUC	0,639	-
Nagashima	Chen FL et al.	375	1-year AUC	0,646	-

		375	3-years AUC	0,593	-
		375	5-years AUC	0,591	-
		424	1-year AUC	0,742	-
		424	3-years AUC	0,653	-
		424	5-years AUC	0,676	-
		296	1-year AUC	0,598	-
		296	3-years AUC	0,646	-
		296	5-years AUC	0,677	-
	Duprè A et al.	219	C-index	0,571	0,522-0,619
Nordlinger	Chen FL et al.	375	1-year AUC	0,619	-
		375	3-years AUC	0,558	-
		375	5-years AUC	0,547	-
		424	1-year AUC	0,625	-
		424	3-years AUC	0,566	-
		424	5-years AUC	0,623	-
		296	1-year AUC	0,546	-
		296	3-years AUC	0,640	-
		296	5-years AUC	0,677	-
	Duprè A et al.	219	C-index	0,563	0,513-0,614
	Skipenko OG et al.	312	3-years C-index	0,600	0,54-0,66
		312	5-years C-index	0,590	0,47-0,71
RASmut-CRS	Brudvik KW et al.	608	p-value	< 0,05	-
	Chen FL et al.	375	1-year AUC	0,739	-
		375	3-years AUC	0,650	-
		375	5-years AUC	0,633	-
		424	1-year AUC	0,674	-
		424	3-years AUC	0,647	-
		424	5-years AUC	0,676	-
		296	1-year AUC	0,712	-
		296	3-years AUC	0,737	-
		296	5-years AUC	0,775	-
	Jiang C et al.	371	C-index	0,616	0,576-0,655
		371	1-year AUC	0,684	-
		371	3-years AUC	0,646	-
		371	5-years AUC	0,661	-
	Sasaki K et al.	2365	C-index	0,550	-
		1205	C-index	0,560	-
	Sasaki K et al.	2376	C-index	0,540	0,52-0,56
	Takeda Y et al.	309	p-value	0,218	-
	Wong GYM et al.	103	C-index	0,600	-
RASmut-CRS with authors's score	Sasaki K et al.	2365	C-index	0,600	-
Rees postop	Skipenko OG et al.	312	3-years C-index	0,630	0,56-0,70
		312	5-years C-index	0,500	0,4-0,6
	Skipenko OG et al.	312	3-years C-index	0,690	0,61-0,75
		312	5-years C-index	0,730	0,62-0,84
Shur radiomics	Chen J et al.	28	C-index	0,590	-
TBS	Fruhling P et al.	1212	C-index	0,580	-

	Sasaki K et al.	430	AIC	1811,000	-
		198	AIC	716,000	-
	Villard C et al.	1013	C-index	0,630	-
Trascriptom	Wada Y et al.	151	p-value	0,006	-
Zakaria's scale	Skipenko OG et al.	312	3-years C-index	0,480	0,42-0,54
		312	5-years C-index	0,600	0,44-0,76
