# Supplementary material for: Prognostic Scores for Liver Resection in Colorectal Metastases: Performance, Limitations, and Methodological Pitfalls—A Systematic Review and Meta-Analysis
Source: Cancers (Basel). 2026 Feb 14;18(4):625. doi: 10.3390/cancers18040625 (PMC12939581; doi:10.3390/cancers18040625)
Supplement: Supplementary file 1 [file cancers-18-00625-s001.zip › Supplementary Table 5.pdf]

Supplementary Table S5. Performances of the score for RFS prediction.

Analyzed score	Author	# patients	Metric	Value	95%CI
Author's immunoscore	Zhang C et al.	106	C-index	0,760	0,659-0,861
		95	C-index	0,715	0,558-0,872
Author's nomogram	Liu W et al.	117	C-index	0,770	-
Author's nomogram	Liu W et al.	237	C-index	0,682	-
		237	1-year AUC	0,700	0,622-0,770
		237	3-years AUC	0,911	0,862-0,961
		237	5-years AUC	0,888	0,815-0,962
Author's random forest model	Chen Q et al.	230	1-year AUC	0,725	-
		230	3-years AUC	0,782	-
		230	5-years AUC	0,792	-
Author's score	Bai L et al.	325	2-years C-index	0,640	0,608-0,672
Author's score	Gasser E et al.	527	p-value	0,011	-
Beppu score	Beppu T et al.	469	C-index	0,560	0,52-0,60
		1756	C-index	0,630	0,61-0,65
		1756	3-years AUC	0,650	-
		1756	5-years AUC	0,650	-
	Kim WJ et al.	295	AUC	0,733	0,665-0,702
	Takematsu T et al.	218	p-value	< 0,001	-
Fong	Bai L et al.	325	2-years C-index	0,598	0,565-0,631
		341	2-years C-index	0,671	0,639-0,703
	Bolhuis K et al.	1105	1-year C-index	0,586	0,564-0,608
		1105	3-years C-index	0,581	0,561-0,602
	Brudvik KW et al.	564	C-index	0,580	0,47-0,68
	Chen Q et al.	389	C-index	0,600	-
		389	1-year AUC	0,650	-
		389	3-years AUC	0,658	-
		389	5-years AUC	0,699	-
	Chen Y et al.	787	C-index	0,586	-
		787	1-year AUC	0,598	-
		787	2-years AUC	0,625	-
		787	33-months AUC	0,601	-
		162	C-index	0,644	-
		162	1-year AUC	0,668	-
		162	18-months AUC	0,780	-
	Katipally RR et al.	147	K-index	0,550	0,49-0,61
	Kim WJ et al.	295	AUC	0,721	0,654-0,789
	Liu W et al.	532	C-index	0,642	-
		237	1-year AUC	0,463	0,391-0,535
		237	3-years AUC	0,499	0,375-0,622

		237	5-years AUC	0,556	0,382-0,730
	Paredes AZ et al.	703	1-year AUC	0,572	0,561-0,582
		703	3-years AUC	0,551	0,542-0,560
		703	5-years AUC	0,551	0,542-0,562
		703	1-year AUC	0,527	0,514-0,538
		703	3-years AUC	0,517	0,504-0,527
		703	5-years AUC	0,516	0,503-0,528
		703	1-year AUC	0,553	0,542-0,562
		703	3-years AUC	0,565	0,554-0,574
		703	5-years AUC	0,555	0,546-0,563
		703	1-year AUC	0,524	0,512-0,539
		703	3-years AUC	0,552	0,543-0,564
		703	5-years AUC	0,548	0,538-0,557
	Wang Y et al.	249	AUC	0,675	0,601-0,749
	Zhai Y et al.	147	AUC	0,570	-
	Zhang C et al.	106	C-index	0,661	-
		95	C-index	0,657	-
	Zhou Z et al.	118	AUC	0,737	-
Fong with molecular subtype	Katipally RR et al.	147	K-index	0,620	0,57-0,67
GAME	Bolhuis K et al.	1105	1-year C-index	0,585	0,561-0,608
		1105	3-years C-index	0,579	0,557-0,600
	Chen Y et al.	787	C-index	0,602	-
		787	1-year AUC	0,608	-
		787	2-years AUC	0,636	-
		787	33-months AUC	0,599	-
		162	C-index	0,691	-
		162	1-year AUC	0,731	-
		162	18-months AUC	0,778	-
	Martin-Cullell B et al.	176	p-value	< 0,05	-
Integrated clinical-molecular group risk	Katipally RR et al.	147	K-index	0,650	0,60-0,70
Iwatsuki	Liu W et al.	237	1-year AUC	0,592	0,518-0,666
		237	3-years AUC	0,562	0,438-0,686
		237	5-years AUC	0,440	0,273-0,607
Modified Glasgow Prognostic score	Furukawa K et al.	149	AUC	0,635	-
Nordlinger	Liu W et al.	237	1-year AUC	0,539	0,459-0,620
		237	3-years AUC	0,586	0,407-0,765
		237	5-years AUC	0,466	0,199-0,733
RASmut-CRS	Paredes AZ et al.	703	1-year AUC	0,525	0,514-0,533
		703	3-years AUC	0,549	0,542-0,555
		703	5-years AUC	0,540	0,533-0,546
		703	1-year AUC	0,501	0,496-0,509

		703	3-years AUC	0,515	0,506-0,524
		703	5-years AUC	0,512	0,503-0,521
		237	1-year AUC	0,529	0,450-0,609
TBS	Liu W et al.	237	3-years AUC	0,543	0,399-0,687
		237	5-years AUC	0,382	0,223-0,541
Trascriptom	Wada Y et al.	151	p-value	< 0,001	-
Trascriptom with clinical score	Wada Y et al.	151	p-value	< 0,001	-
